# Aerobic exercise alleviates ventilator-induced lung injury by inhibiting NLRP3 inflammasome activation

**DOI:** 10.1186/s12871-022-01874-4

**Published:** 2022-12-01

**Authors:** Mengjie Liu, Yaqiang Zhang, Jie Yan, Yuelan Wang

**Affiliations:** 1 Department of Anesthesiology and Perioperative Medicine, Shandong Provincial Qianfoshan Hospital, Shandong University, 250012 Jinan, Shandong China; 2 Department of Anesthesiology and Perioperative Medicine, Shandong Institute of Anesthesia and Respiratory Intensive Care Medicine, The First Affiliated Hospital of Shandong First Medical University, 250014 Jinan, Shandong China; 3 Beijing Sport University, Xinxi Road, Haidian District, 100084 Beijing, China; 4 Department of Anesthesiology and Perioperative Medicine, Shandong Provincial Qianfoshan Hospital, Shandong University, No.16766, Jingshi Road, 250014 Jinan, China

**Keywords:** Aerobic exercise, Ventilator-induced lung injury, NLRP3 inflammasome

## Abstract

**Background:**

Ventilator-induced lung injury (VILI) is caused by stretch stimulation and other factors related to mechanical ventilation (MV). NOD-like receptor protein 3 (NLRP3), an important innate immune component, is strongly associated with VILI. This study aimed to investigate the effect and mechanisms of aerobic exercise (EX) on VILI.

**Methods:**

To test the effects of the PKC inhibitor bisindolylmaleimide I on PKC and NLRP3, male C57BL/6 mice (7 weeks old, 19 ~ 23 g) were randomly divided into four groups: control group(C), bisindolylmaleimide I-pretreated group(B), MV group, and bisindolylmaleimide I-pretreated + MV (B + MV) group. The mice were pretreated with bisindolylmaleimide I through intraperitoneal injection (0.02 mg/kg) 1 h before MV. MV was performed at a high tidal volume (30 ml/kg). To explore the ameliorative effect of EX on VILI, the mice were randomly divided into C group, MV group, EX group and EX + MV group and subjected to either MV or 5 weeks of EX training. After ventilation, haematoxylin-eosin (HE) staining and wet/dry weight ratio was used to assess lung pathophysiological changes. PKCɑ, P-PKCɑ, ASC, procaspase-1, caspase-1, pro-IL-1β, IL-1β, NLRP3 and occludin (tight junction protein) expression in lung tissues was determined by Western blotting. The level of IL-6 in alveolar lavage fluid was determined by ELISA.

**Results:**

NLRP3, P-PKCɑ, and PKCɑ levels were inceased in MV group, but bisindolylmaleimide I treatment reversed these changes. Inhibition of PKC production prevented NLRP3 activation. Moreover, MV increased ASC, procaspase-1, caspase-1, pro-IL-1β, and IL1β levels and decreased occludin levels, but EX alleviated these changes. HE staining and lung injury scoring confirmed an absence of obvious lung injury in C group and EX group. Lung injury was most severe in MV group but was improved in EX + MV group. Overall, these findings suggest that MV activates the NLRP3 inflammasome by activating PKCɑ and inducing occludin degradation, while Exercise attenuates NLRP3 inflammasome and PKCɑ activation. Besides, exercise improves cyclic stretch-induced degradation of occludin.

**Conclusion:**

PKC activation can increase the level of NLRP3, which can lead to lung injury. Exercise can reduce lung injury by inhibiting PKCɑ and NLRP3 activation. Exercise maybe a potential measure for clinical prevention of VILI.

**Supplementary Information:**

The online version contains supplementary material available at 10.1186/s12871-022-01874-4.

## Background

Acute lung injury (ALI) is a common clinical respiratory illness caused by a variety of noncardiac factors. The main manifestations are injury to pulmonary capillary endothelial cells and alveolar epithelial cells and increased vascular permeability, which can develop into acute respiratory distress syndrome (ARDS). The characteristics of ARDS are acute and progressive dyspnoea, refractory hypoxemia and pulmonary edema [1]. Mechanical ventilation is important in the treatment of ALI and ARDS, but it may aggravate lung injury and lead to ventilator-induced lung injury (VILI). The exact mechanisms underlying VILI remain unknown. The associated mechanical stretch can lead to excessive alveolar expansion, increased intrapulmonary pressure, pressurized pulmonary edema, and stimulation of inflammatory cytokine release, thus leading to inflammatory injury.

NOD-like receptor protein 3 (NLRP3) is an important component of innate immunity and is strongly associated with the occurrence of VILI. Previous studies have shown that cyclic stretch activates the NLRP3 inflammasome by activating NIMA-related kinase 7 (NEK7) [2]. By inhibiting the toll-like receptor 4 (TLR4) pathway and ROS production, p120 can prevent the activation of NLRP3 [1, 3]. Our previous research also found that mechanical ventilation could activate the PKC signalling pathway and that upregulation of occludin can reduce VILI. Multiple studies have shown that exercise can improve lung function and reduce lung injury in patients through a variety of mechanisms. For example, aerobic exercise alleviates ALI through regulating neutrophil extracellular traps [4]. Moreover, exercise can modulate the balance of inflammatory/anti-inflammatory and oxidative/antioxidative processes in the early stage of ALI [5]. Some studies have also shown that exercise can alleviate Alzheimer’s disease and diet-induced nonalcoholic steatohepatitis by inhibiting NLRP3 inflammasome activation [6]. However, it remains unknown whether aerobic exercise affects VILI and whether NLRP3 or PKCɑ participates in this process. Therefore, the relationship among PKCɑ, NLRP3 and aerobic exercise in VILI should be further explored.

In this study, we established a mouse model of aerobic exercise in VILI to explore the protective role of aerobic exercise and the mechanisms of NLRP3 in VILI. We observed that PKCɑ upregulated NLRP3 activation, whereas 5 weeks of aerobic exercise inhibited the activation of NLRP3, thereby reducing lung injury. All our findings suggest that exercise may be a new direction for the prevention of VILI.

## Materials and methods

### Animals and treatment

Seven-week-old male C57BL/6 N mice weighing 19–23 g were supplied by the Experimental Animal Center of Shandong University and fed for 1 week to allow them to adapt to the environment. The mice were kept in a constant temperature of 25 °C under a 12-hour light/dark cycle and were fed a standard diet of pellets and water.

To determine the relationship between PKC activation and NLRP3, the mice were randomly divided into the following four groups (n = 6 in each group): control (C) group, bisindolylmaleimide I-pretreated (0.02 mg/kg)(B) group, mechanical ventilation (MV) group and bisindolylmaleimide I-pretreated (1 h) and mechanically ventilated (B + MV) group. Mechanical ventilation was not conducted in C and B groups. The other two groups were mechanically ventilated for 4 h using an ALC-V8 animal ventilator. The ventilation parameters were set as follows: tidal volume 30 ml/kg, respiratory rate 60 times/min, I/E ratio of 1:2, no positive end-expiratory pressure, fraction of inspired oxygen 21% and room temperature 25 °C.

For another study, the mice were randomly divided into the following four groups (n = 6 in each group): control (C) group, mechanical ventilation (MV) group, exercise (EX) group (which only underwent 5 weeks of aerobic exercise training), and exercise + mechanical ventilation (EX + MV) group (which underwent 5 weeks of aerobic exercise plus mechanical ventilation). The ventilation parameters were set as described above [7]. All experimental protocols and mouse research procedures involved in this study were approved by the Ethics Committee of Shandong Provincial Qianfoshan Hospital. The experimental data were based on three repeated experiments.

### Aerobic exercise protocol

Mice were subjected to mechanical ventilation or 5 weeks of aerobic exercise training. The specific aerobic exercise regimen was as follows: mice were acclimated to a treadmill for 3 days (15 min per day, 25% slope, 0.2 km/h). The maximum aerobic capacity of the mice was determined, and the treadmill speed was increased by 0.1 km/h every 2.5 min until exhaustion (defined as the inability of the mice to run after 10 mechanical stimuli). The maximum speed each mouse achieved during treadmill training was recorded as the maximum aerobic capacity (100%). Mice in EX group and EX + MV group received aerobic exercise training at a moderate aerobic exercise capacity (50%) for 60 min a day, 5 days a week for 5 weeks^[[4, 5]]^. Mice in C group and MV group were raised in standard cages for 5 weeks without intervention.

### Reagents and instruments

Shanghai Alcott Biotech Co. (Shanghai, China) provided the ALC-V8 animal ventilator. The animal experiments platform ZH-PT/5S was purchased from Guizhou Zhenghua Biological Instrument Co., Ltd. (Guizhou, China). Enzyme-linked immunosorbent assay (ELISA) kits for interleukin (IL)-1β and IL-6 were purchased from Elabscience Biotechnology Co., Ltd. (Wuhan, China). Rabbit anti-mouse NLRP3, GAPDH and ɑ-tubulin antibodies were purchased from Cell Signaling Technology, Inc. (CST, USA). Antibodies against ASC, procaspase-1, caspase-1, and P-PKC α were purchased from Santa Cruz, Inc. (Santa Cruz, USA). Antibodies against IL-1β, pro-IL-1β, PKCα, and occludin were purchased from Abcam, Inc. (Abcam, USA). A bicinchoninic acid kit, radio immunoprecipitation assay lysis buffer and protease inhibitor were purchased from Shanghai Biyuntian Biological Company (Shanghai, China). A haematoxylin-eosin (HE) staining kit was purchased from Beijing Zhongshan Biotechnology Co., Ltd. (Beijing, China).

### Experimental protocol and sample harvesting

Mice in C, B and EX groups were sacrificed after tracheotomy, while the other four groups were sacrificed after ventilation (4 h). Mice were given 150 mg/kg sodium pentobarbital to keep them under deep anaesthesia and then sacrificed by exsanguination through the abdominal aorta. Arterial blood was quickly harvested from the four groups. Western blotting was performed on the superior lobe of the right lung, and the remaining right lung tissue was fixed for HE staining. The other lung was used to calculate the lung wet/dry (W/D) weight ratio [8].

### Lung wet/dry (W/D) weight ratio

After mechanical ventilation, lung tissues were collected and washed rapidly, then the wet lung weight was determined. After that, the lung tissue was heated to measure dry weight (70 °C for 72 h). Then, we calculated the pulmonary W/D ratio to evaluate the degree of pulmonary edema.

### Collection of bronchoalveolar lavage fluid (BALF)

Mice were tracheotomized and cannulated with a 20-gauge catheter. The lungs were lavaged three times with 1.0 ml of ice-cold D-PBS to collect BALF. Then, the BALF was centrifuged at 1500 rpm at -4 °C for 5 min, and the supernatant was collected and stored at -20 °C for ELISA detection.

### Enzyme-linked immunosorbent assay (ELISA)

The levels of IL-6 were determined according to the ELISA kit instructions (Elabscience, China).

### Haematoxylin-eosin (HE) staining and lung injury scoring

In brief, the lung tissue was fixed with formalin, embedded in paraffin, sectioned and observed under a 400× light microscope. Previous studies have introduced criteria for evaluating pulmonary pathological changes. The criteria required at least three visual fields to be observed for each slice. Pulmonary structure destruction, pulmonary edema, alveolar septum thickening, hyaline membrane formation, alveolar haemorrhaging and inflammatory cell infiltration were evaluated. The severity of lung injury was described on a scale of 0–4, where 0 indicates no injury, 1 indicates mild injury, 2 indicates moderate injury, 3 indicates severe injury, and 4 indicates serious injury. The cumulative total score was the final lung injury score [1, 9].

### Western blot analysis

The tissue fragments were lysed in radioimmunoprecipitation assay buffer and then centrifuged for 5 min at 12,000 × g. The lysate was collected, and the protein concentration was measured using a test kit. Equal amounts of protein were denatured and separated on 10% SDS‒PAGE gels and then transferred to polyvinylidene difluoride membranes. When we performed WB, the blots were cut prior to hybridization with antibodies, and we developed strips of different proteins on the same PVDF film separately. The membranes were blocked in 5% nonfat milk for 2 h at room temperature and subsequently incubated with primary antibodies overnight at 4 °C. The primary antibodies targeted the following proteins: NLRP3 (1:500), ɑ- tubulin (1:1000), GAPDH (1:3000) (Cell Signaling Technology, USA), ASC (1:200), procaspase-1, caspase-1 (1:500), p-PKC α (1:500) (Santa Cruz, USA), IL-1β, pro-IL-1β (1:1000), PKC α (1:1000), and occludin (1:2000) (Abcam, USA). Then, the membranes were incubated with secondary antibodies (1:5000) for 2 h at room temperature, and ECL Super Signal reagent (Millipore, USA) was used to detect protein bands. Image J software was used to measure the relative band density of different proteins on the scanned membrane [1, 8].

### Statistical analysis

Statistical analysis was performed using SPSS 22 and Prism 8. All the results are expressed as the mean ± SD. The unpaired T test or one-way analysis of variance (ANOVA) was used for Gaussian data, and the Mann‒Whitney U test or Wilcoxon rank sum test was used for nonparametric data. P ≤ 0.05 was considered to indicate statistical significance.

## Results

### Mechanical ventilation enhanced NLRP3 level through PKC activation

**Fig. 1:**
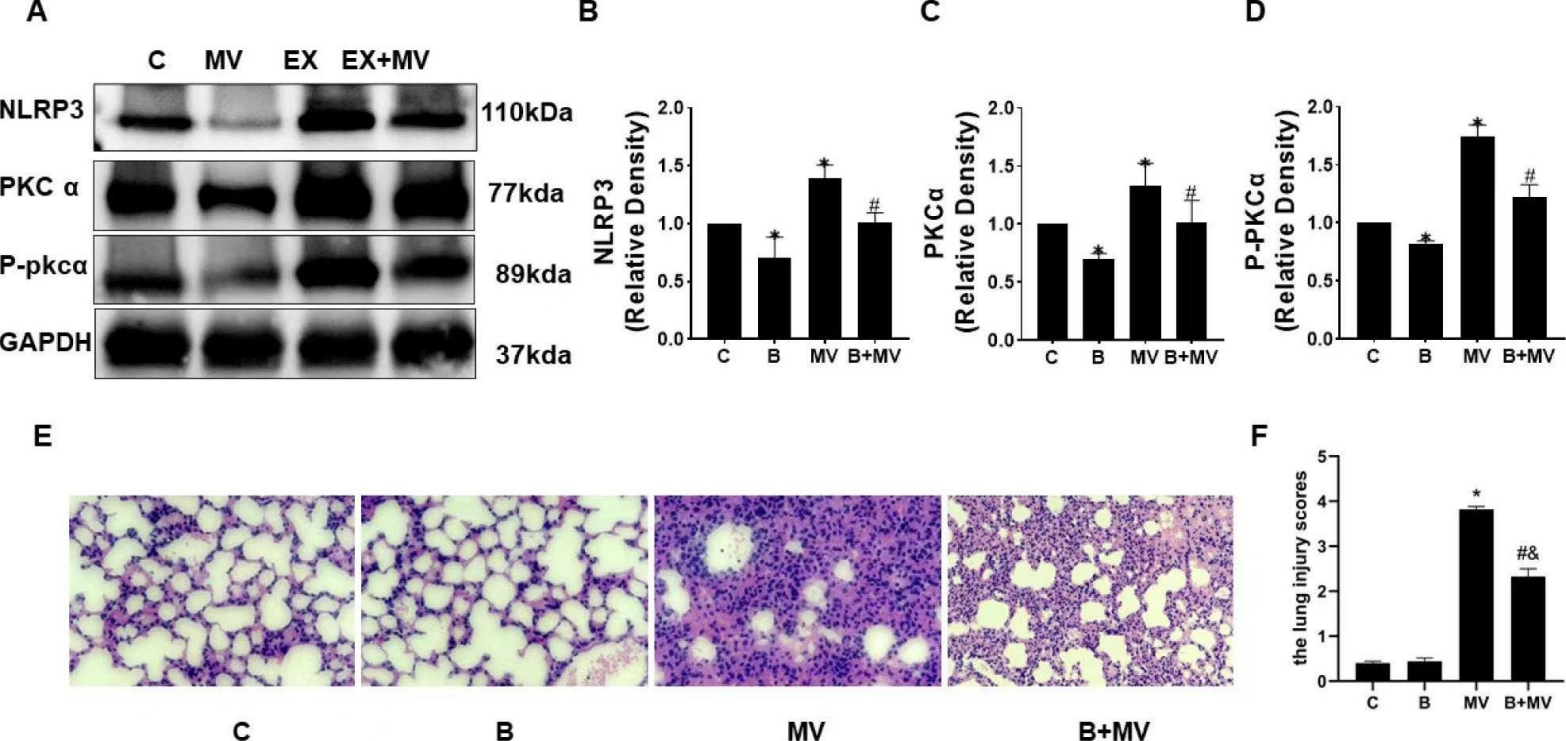
Mechanical ventilation increased the levels of NLRP3, P-PKCɑ, and PKCɑ, but these changes were reversed by bisindolylmaleimide I. Inhibition of PKC expression prevented the activation of NLRP3. (A) The protein levels of NLRP3, PKCɑ and P-PKCɑ were measured by Western blotting. When we performed WB, the blots were cut prior to hybridization with antibodies, and we developed strips of different proteins on the same PVDF film separately. The unedited blots are shown in the Supplementary File. (B, C, D) *p < 0.01 versus the C group, #p < 0.01 versus the MV group. C, control group; B, group pretreated with bisindolylmaleimide I 0.02 mg/kg MV, mechanical ventilation group; B + MV, group pretreated with bisindolylmaleimide I 0.02 mg/kg for 1 h before mechanical ventilation (4 h). (E-F) The pathological changes in lung tissues were determined by HE staining (magnification 400×) and lung injury scores. *p < 0.01 versus the C group, ^*#*^*p < 0.01* versus the B group, and ^&^*p < 0.01* versus the MV group. The scale bar represents 16 μm. The data are representative of three independent experiments

To determine the relationship between PKC and NLRP3 in VILI model, mice were pretreated with the PKC inhibitor bisindolylmaleimide I and then treated with mechanical ventilation. NLRP3, P-PKCɑ and PKCɑ were determined by Western blot analysis. The results suggested that NLRP3, P-PKCɑ and PKCɑ expression were higher in MV group than those in C group (*p < 0.01*) (Fig. 1A), but the increases were alleviated by bisindolylmaleimide I pretreatment in B + MV group (*p < 0.01*) (Fig. 1A–D). Compared with that in C and B + MV groups, the expression of PKC and P-PKC was significantly lower in B group, which verified the specific inhibitory effect of bisindolylmaleimide I on PKC. In addition, the expression of NLRP3 was decreased. The HE staining results were consistent with the Western blot results. As shown in Fig. 1E-F, compared with C group, no obvious lung injury was found in B group, while pulmonary edema, inflammatory cell infiltration were showed in MV group. And lung injury was alleviated in B + MV group compared with MV group. All of these results indicated that mechanical ventilation induced the activation of PKC and NLRP3, while PKC inhibitor treatment significantly inhibited the activity of PKC α and P-PKCα, which lead to the decrease of NLRP3. Thus, the high level of NLRP3 maybe caused by PKC activation induced by MV.

### Aerobic exercise inhibited the activity of the NLRP3 inflammasome induced by mechanical ventilation

**Fig. 2:**
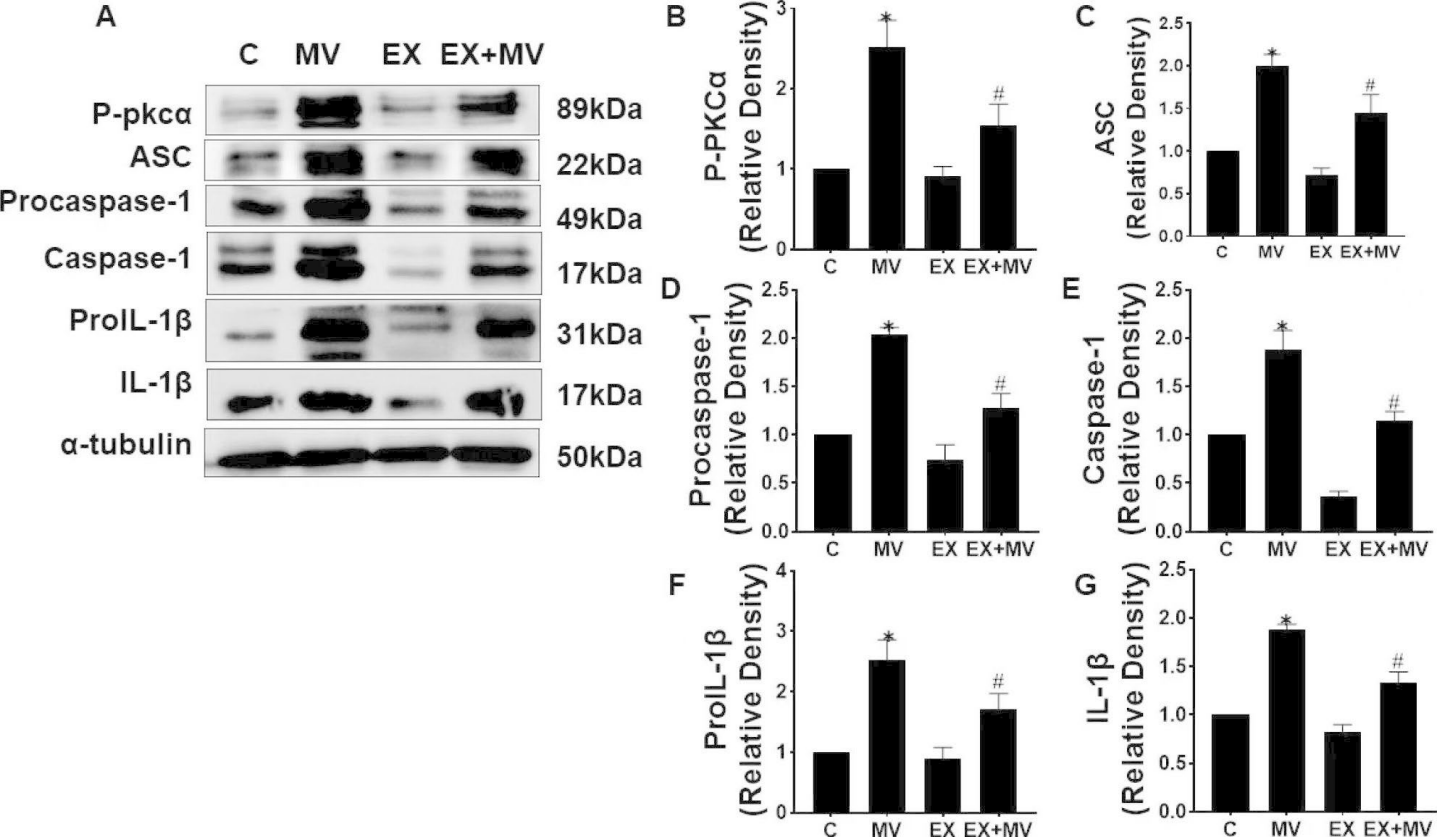
Aerobic exercise inhibited the assembly and activity of the NLRP3 inflammasome induced by mechanical ventilation. (A) The protein levels of P-PKCɑ, ASC, procaspase-1, caspase-1, pro-IL-1β and IL-1β were detected by Western blotting. When we performed WB, the blots were cut prior to hybridization with antibodies, and we developed strips of different proteins on the same PVDF film separately. The unedited blots are shown in the Supplementary File. (B-G) *p < 0.01 versus the C group, #p < 0.01 versus the MV group. C, control group; MV, mechanical ventilation group; EX, exercise group (only received 5 weeks of aerobic exercise training); EX + MV, exercise + mechanical ventilation group (underwent 5 weeks of aerobic exercise running plus mechanical ventilation). The data are representative of three independent experiments

We determined the impact of aerobic exercise on NLRP3 inflammasome activation. The expression of P-PKCɑ, ASC, procaspase-1, caspase-1, pro-IL-1β and IL-1β were detected by Western blotting. NLRP3, ASC and procaspase-1 assemble into the NLRP3 inflammasome. We found that the expression of P-PKCɑ, ASC, procaspase-1, and caspase-1 was obviously increased after mechanical ventilation; however, this effect was alleviated by aerobic exercise treatment. Therefore, we concluded that mechanical ventilation clearly induced the activity of the NLRP3 inflammasome and PKC. Pro-IL-1β and IL-1β release relied on NLRP3 inflammasome activation in response to mechanical ventilation, which was also affected by aerobic exercise. Compared with that in C group, the expression of P-PKCɑ, ASC, procaspase-1, caspase-1, pro-IL-1β and IL-1β in EX group were slightly but not significantly lower. However, compared with that in the MV group, the expression of these proteins was significantly lower in aerobic exercise pretreatment group (*p < 0.*01, Fig. 2).

### Aerobic exercise ameliorates ventilator-induced activation of NLRP3 inflammasome

**Fig. 3:**
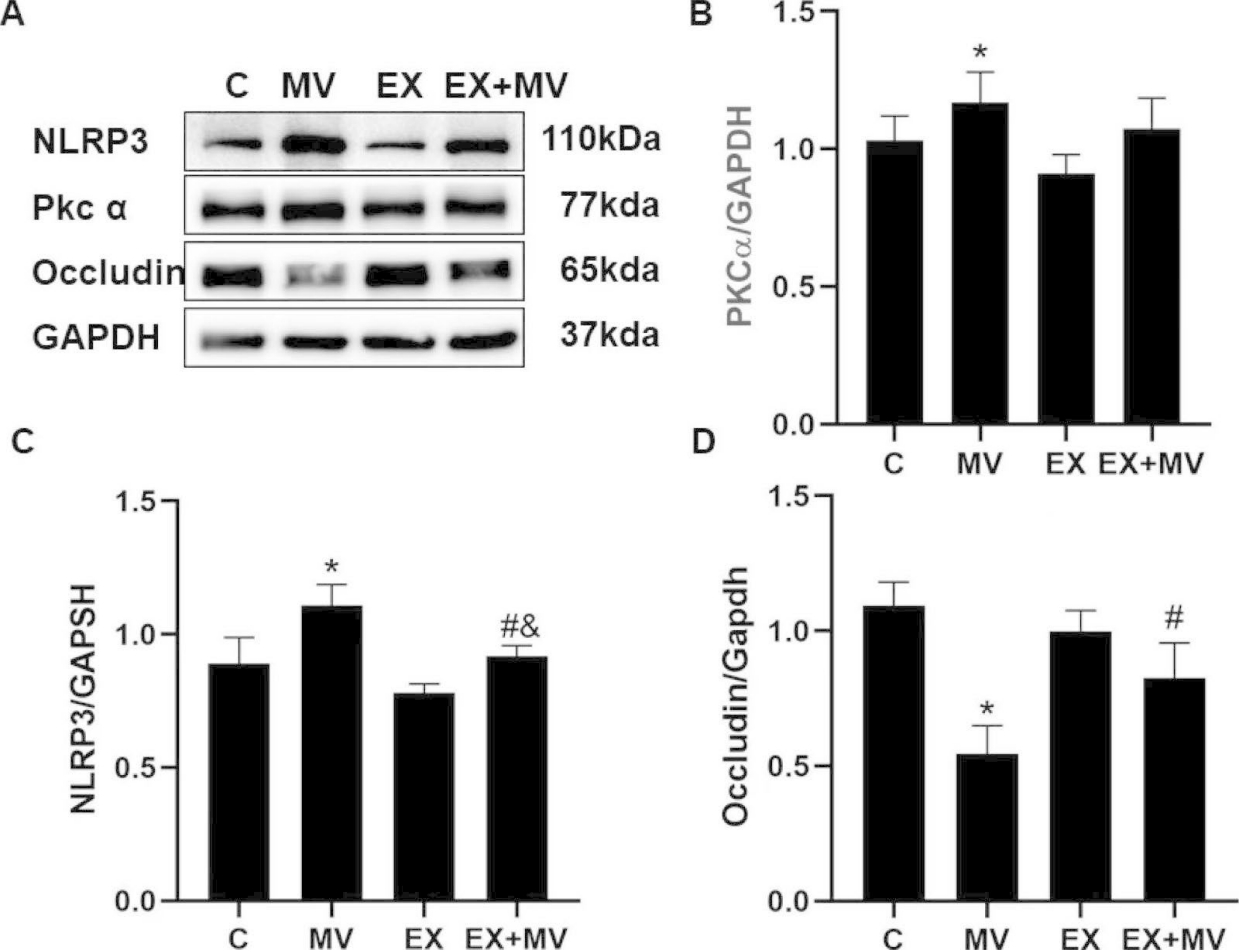
NLRP3 regulated the activation of the NLRP3 inflammasome as well as the degradation of cell junction proteins after mechanical ventilation, but aerobic exercise alleviated these changes. (A) Western blotting was used to determine the expression of NLRP3, PKCɑ and occludin. GAPDH was used as a reference. When we performed WB, the blots were cut prior to hybridization with antibodies, and we developed strips of different proteins on the same PVDF film separately. The unedited blots are shown in the Supplementary File. (B) *p < 0.01 versus the C group, #p < 0.01 versus the MV group, &p < 0.01 versus the EX group. C, control group; MV, mechanical ventilation group; EX, 5-week aerobic training intervention group; EX + MV, 5-week aerobic training intervention plus mechanical ventilation group. The data are representative of three independent experiments

The level of NLRP3, PKCɑ and occludin were detected by Western blotting. The level of NLRP3 was significantly higher in the mechanical ventilation group than in the C group and EX group, while NLRP3 expression was significantly lower in the EX + MV group than in the MV group (*p < 0.01*, Fig. 3A, C). This finding indicated that mechanical ventilation activated the NLRP3 inflammasome and increased the expression of NLRP3, which was ameliorated by aerobic exercise. And the level of PKCɑ increased in MV group while decreased in EX group. (Fig. 3A, B). Activation of NLRP3 caused by mechanical ventilation further induced degradation of occludin (*p < 0.01*). However, the degradation of occludin was alleviated in EX + MV group (5 weeks excise before ventilation for 4 h) (*p < 0.01*) (Fig. 3A, D).

### Aerobic exercise suppressed IL-6 production induced by mechanical ventilation

**Fig. 4:**
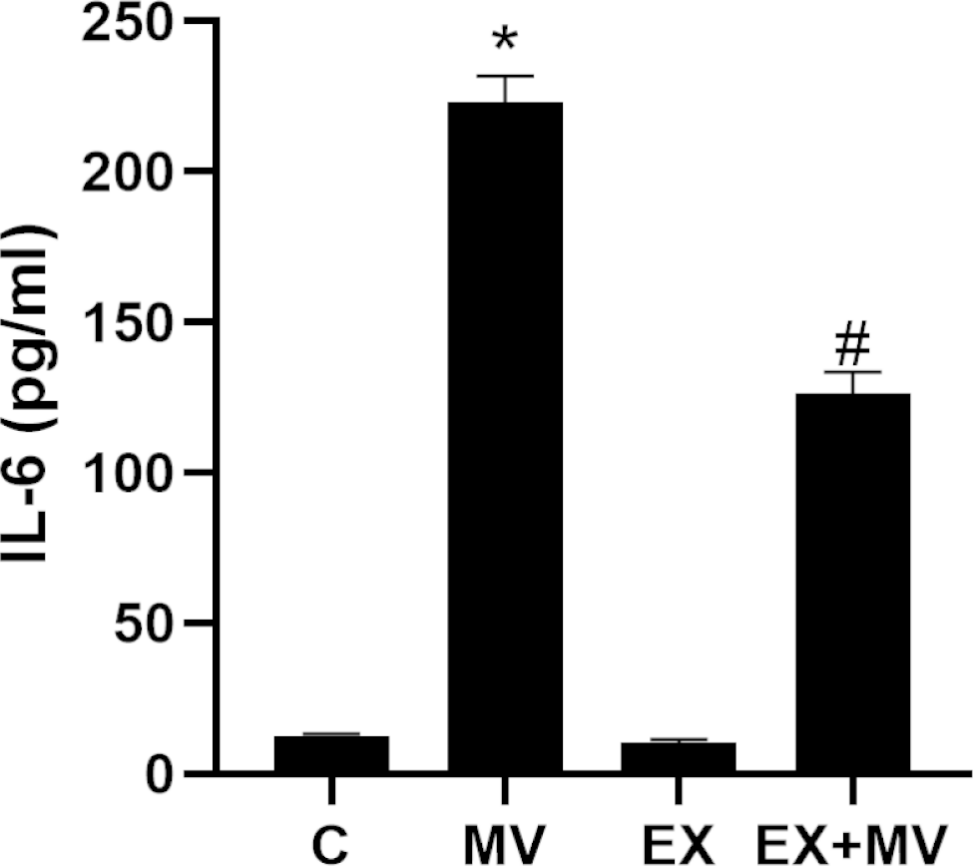
Role of aerobic exercise in regulating the secretion of IL-6 caused by mechanical ventilation. The levels of IL-6 in BALF were detected by ELISA. All experiments were repeated at least three times. *p < 0.01 versus the C group, ^#^*p < 0.01* versus the MV group

Mechanical ventilation induced NLRP3 inflammasome activation and IL-6 production. IL-6 levels in BALF were detected by ELISA, as shown in Fig. 4. The IL-6 levels were significantly higher in MV group and EX + MV group than in C group; however, the level was significantly lower in EX + MV group than in MV group (p < 0.001) (Fig. 4).

### Activation of NLRP3 exacerbated lung injury, while aerobic exercise alleviated lung injury and pulmonary edema caused by mechanical ventilation

**Fig. 5:**
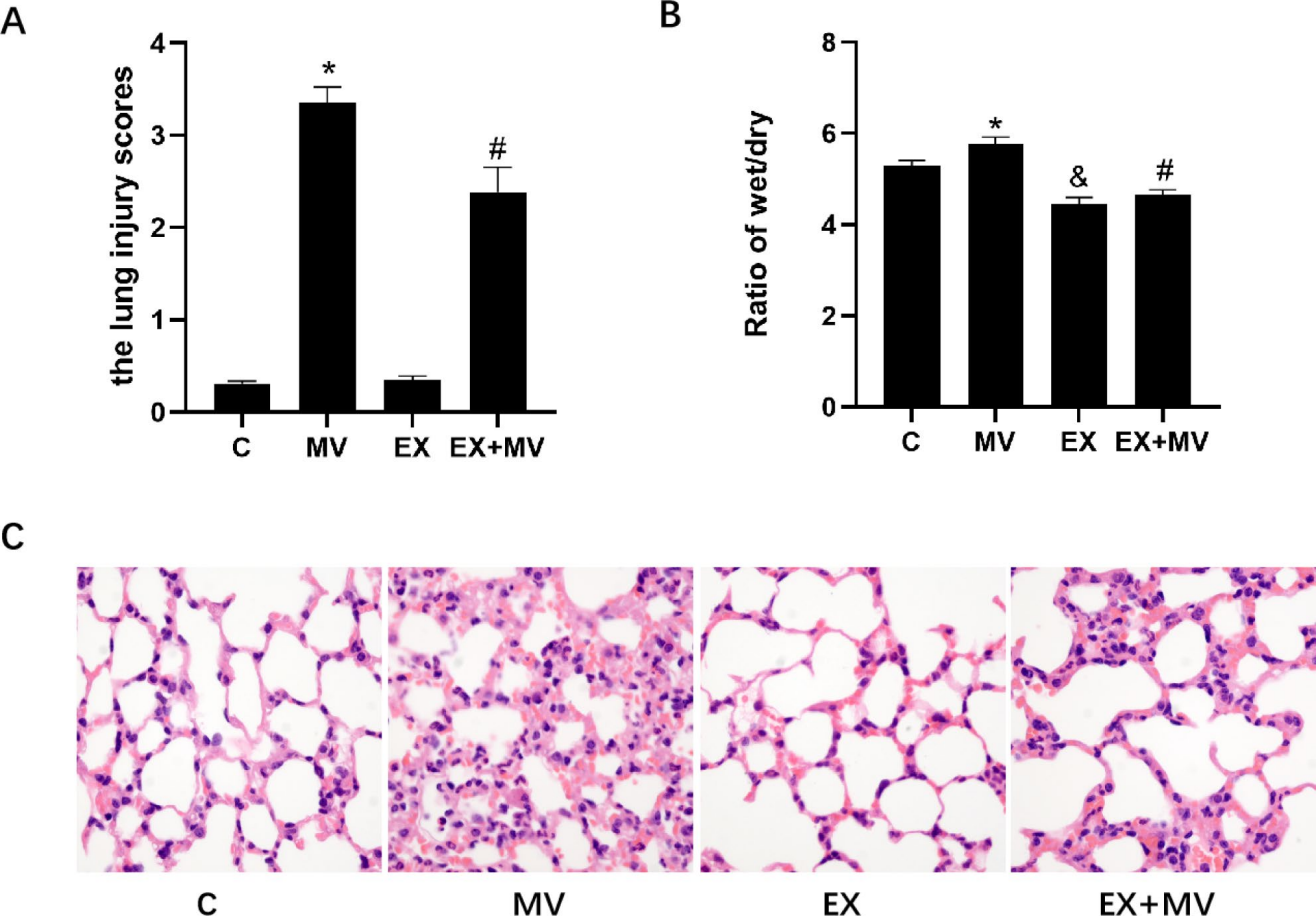
Role of NLRP3 in VILI and the effect of aerobic training intervention. (A) The degree of pulmonary edema was evaluated by the lung W/D ratio. *p < 0.01 versus the C group, ^*#*^*p < 0.01* versus the MV group. (B) Lung injury scores were applied to evaluate the severity of lung injury in different groups. *p < 0.01 and ^&^*p < 0.01* versus the C group, ^*#*^*p < 0.01* versus the MV group. (C) The pathological changes in lung tissues were determined by HE staining (magnification 400×). The scale bar represents 16 μm. The data are representative of three independent experiments

HE staining was used to assess the pathological changes in lung tissues. As shown in Fig. 5C, pulmonary alveolar haemorrhage and edema, inflammatory cell infiltration and lung tissue structure destruction were observed in mice in MV group. The above phenomena were alleviated in EX + MV group, and only a few infiltrating inflammatory cells were found in C group and EX groups. In addition, the lung injury scores of each group were in good concordance with the above results (Fig. 5B).

The degree of pulmonary edema was expressed by the lung W/D ratio. As shown in Fig. 5A, the W/D ratios in EX group and EX + MV group were lower, while the W/D ratio of the MV group was significantly higher than that of C group (p < 0.01). However, aerobic exercise pretreatment significantly reduced the lung W/D ratio in EX + MV group compared with MV group (p < 0.01).

## Discussion

**Fig. 6:**
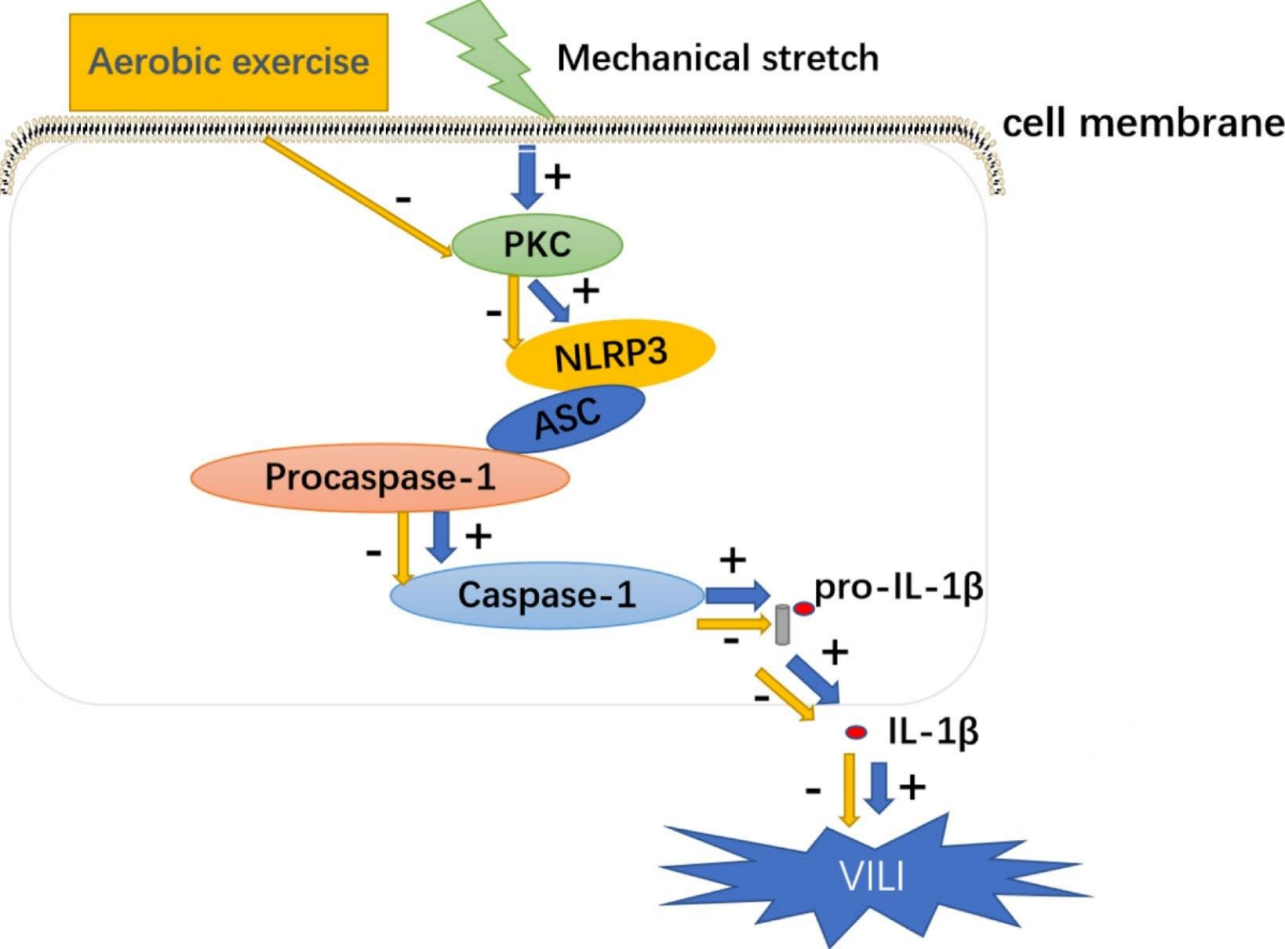
Mechanisms of aerobic exercise and NLRP3-related regulation of ventilator-induced lung epithelial injury

ALI is a common clinical respiratory condition caused by a variety of noncardiac factors, and further deterioration can lead to ARDS [10–12]. Mechanical ventilation is an important treatment for ALI/ARDS, but it may aggravate lung injury and lead to VILI. A large number of local or systemic inflammatory factors are released during VILI, and the NLRP3 inflammasome is one of the most important mediators of inflammation [13, 14] (Fig. 6).

Previous studies have shown that cyclic stretch induces NLRP3 inflammasome activation by activating NEK7, resulting in mitochondrial dysfunction, while p120 has a protective effect on mitochondria by inhibiting NLRP3 activation [2, 3, 15]. An et al. confirmed that oxidative stress may lead to VILI by activating the NLRP3 inflammasome and TRPM2 channels [16]. Therefore, this study focused on the role of NLRP3 in lung injury caused by mechanical ventilation. Previous studies have mainly focused on mechanisms without investigating prevention measures, but some research has suggested that aerobic exercise can inhibit the inflammatory response [17]. For example, one study showed that aerobic exercise alleviates ALI through neutrophil extracellular trap-induced alveolar macrophage proinflammatory polarization [4]. Therefore, considering our previous studies, we further investigated whether aerobic exercise can play a protective role in lung injury by inhibiting activation of the NLRP3 inflammasome.

Aerobic exercise can improve cardiopulmonary endurance by enhancing myocardial contractility, increasing conduction velocity and cardiac output, inhibiting airway inflammation and improving oxyhaemoglobin saturation to enhance respiratory function [18, 19]. Aerobic exercise can also modulate the inflammatory/anti-inflammatory and oxidative/antioxidative balance in the early stage of LPS-induced lung injury, which leads to a decrease in the release of inflammatory mediators such as TNF-α, IL-6, IL-10, IL-1β, IL-17, GM-CSF, and CXCL1/KC [17, 19]. Research has suggested that treadmill training can reduce lung injury caused by particulate matter (PM) 2.5 exposure by inhibiting the TLR4/NF-κB signalling pathway [20]. Most related studies have focused on the effect of aerobic exercise on lipopolysaccharide (LPS)-induced lung injury, while there have few studies on mechanical ventilation-induced lung injury. Our previous study found that mechanical ventilation can induce PKCα activation [8], and there is also evidence that aerobic exercise inhibits the PKCα/Cav1.2 pathway [21]. Therefore, in this study, we explored the effect and mechanisms of PKC activition on NLRP3 in VILI.

In this study, we first used the PKC inhibitor bisindolylmaleimide I to determine the relationship between PKC and NLRP3. We found that mechanical ventilation induced the activation of PKC and NLRP3, while PKC inhibitor treatment significantly inhibited the activity of PKCα and P-PKCα and decreased the expression of NLRP3. Therefore, we speculated that PKC may promote NLRP3 activation, but the specific mechanism was not clear. The beneficial (inhibitory) influence of aerobic exercise on NLRP3 or PKCα has been reported previously [22], but the relationship between NLRP3 and PKCα needs further elucidation. We hypothesize that PKC acts as a protein kinase and activates subsequent inflammatory signalling pathways, including the NLRP3 pathway.

To determine the effects and mechanisms by which mechanical ventilation and aerobic exercise on NLRP3, mice were divided into four groups, and the expression of P-PKCɑ, ASC, procaspase-1, caspase-1, pro IL-1β and IL-1β were examined. We found that the expression of ASC, procaspase-1, and caspase-1 were obviously increased in the MV group; in contrast, these changes were alleviated by aerobic exercise pretreatment. These findings indicate that mechanical ventilation and aerobic exercise may play opposing roles by influencing NLRP3 inflammasome formulation [3, 23]. The expression of pro-IL-1β and IL-1β relied on the activation of NLRP3, so there were different expression levels in the mechanical ventilation and aerobic exercise groups. These findings are similar to those of previous studies [4, 24].

Adherens proteins and tight junction proteins play vital roles in maintaining normal alveolar permeability [1, 25]. Mechanical stretch induced the degradation of tight junction proteins such as occludin, potentially leading to pulmonary edema; however, the degradation of occludin was decreased in EX + MV group, in which NLRP3 was downregulated by aerobic exercise (Fig. 3). Based on this finding, we suggested that the degradation of occludin induce the enhanced vascular permeability in VILI, possibly due to the activation of NLRP3 inflammasome. However, the specific mechanism need further exploration.

Interleukins play important roles in the maturation, activation, proliferation and immune regulation of immune cells [26]. A previous study revealed that patients with ARDS exhibit elevated levels of IL-6, which correlate with increased morbidity and mortality, and in vitro data showed that binding of IL-6 with its soluble receptor significantly increases endothelial cell permeability [27]. Therefore, in this study, ELISA was used to detect the IL-6 production in BALF. We found that the IL-6 levels were significantly increased in the MV group, but the increase was milder in the MV + EX group.

Noncardiogenic pulmonary edema is the most visible manifestation of VILI. Its fundamental causes are destruction of alveolar membrane integrity and increased pulmonary capillary permeability [28, 29]. In this study, we used HE staining and lung injury scores to assess the pathological changes in lung tissues and the lung W/D ratio to describe the extent of pulmonary edema in a mouse model of VILI and aerobic exercise. Mechanical ventilation increased the lung W/D ratio and lung injury score, which further aggravated the permeabilization of the pulmonary vascular barrier and pulmonary edema. Pulmonary edema of VILI was markedly improved in mice that were mechanically ventilated after 5 weeks of aerobic exercise. These results show that exercise can reduce lung injury caused by mechanical ventilation. Furthermore, the lung W/D ratio of mice in aerobic exercise training group were even lower than mice in control group, which confirms that aerobic exercise can protect the lung function of mice and reduce pulmonary edema and lung injury.

Mechanical ventilation is a double-edged sword; it is often used as a life support to improve oxygen supply in critically ill patients, but its long term use or inappropriate parameter settings may inhibit pulmonary circulation and induce VILI [30, 31]. However, the pathogenesis of VILI is complex and has not yet been completely defined; therefore, the relationship among mechanical ventilation, aerobic exercise and VILI needs further study.

## Conclusion

Taken together, our findings demonstrate that aerobic exercise for 5 consecutive weeks can reduce the induction of lung injury by mechanical ventilation. In terms of the mechanisms, we found that activation of PKC and NLRP3 can lead to ALI. Specific and highly efficient PKC inhibition and aerobic exercise can reduce lung injury by inhibiting the activation of PKCɑ and NLRP3, suggesting a potential measure for the clinical prevention and treatment of VILI.

## Electronic supplementary material

Below is the link to the electronic supplementary material.

Supplementary Material 1

## Data Availability

WB raw data are deposited in the Supplementary Files. The datasets used and/or analyzed during the current study are not publicly available due to data protection but are available from the corresponding author on reasonable request.

## List of abbreviations

ALI: Acute lung injury
ARDS: Acute respiratory distress syndrome
BALF: Bronchoalveolar lavage fluid
ELISA: Enzyme-linked immuno sorbent assay
HE staining: Hematoxylin-eosin staining
NLRP3: NOD-like receptor protein 3
NEK7: NIMA-related kinase 7
PKCɑ: protein kinase C ɑ
VILI: Ventilator-induced lung injury
W/D: Wet/dry weight ratio
