# Supplementary material for: Aerobic exercise alleviates ventilator-induced lung injury by inhibiting NLRP3 inflammasome activation
Source: BMC Anesthesiol. 2022 Dec 1;22:369. doi: 10.1186/s12871-022-01874-4 (PMC9714243; doi:10.1186/s12871-022-01874-4)
Supplement: Supplementary file 1 — Supplementary Material 1 [file 12871_2022_1874_MOESM1_ESM.pptx]

## Slide 1
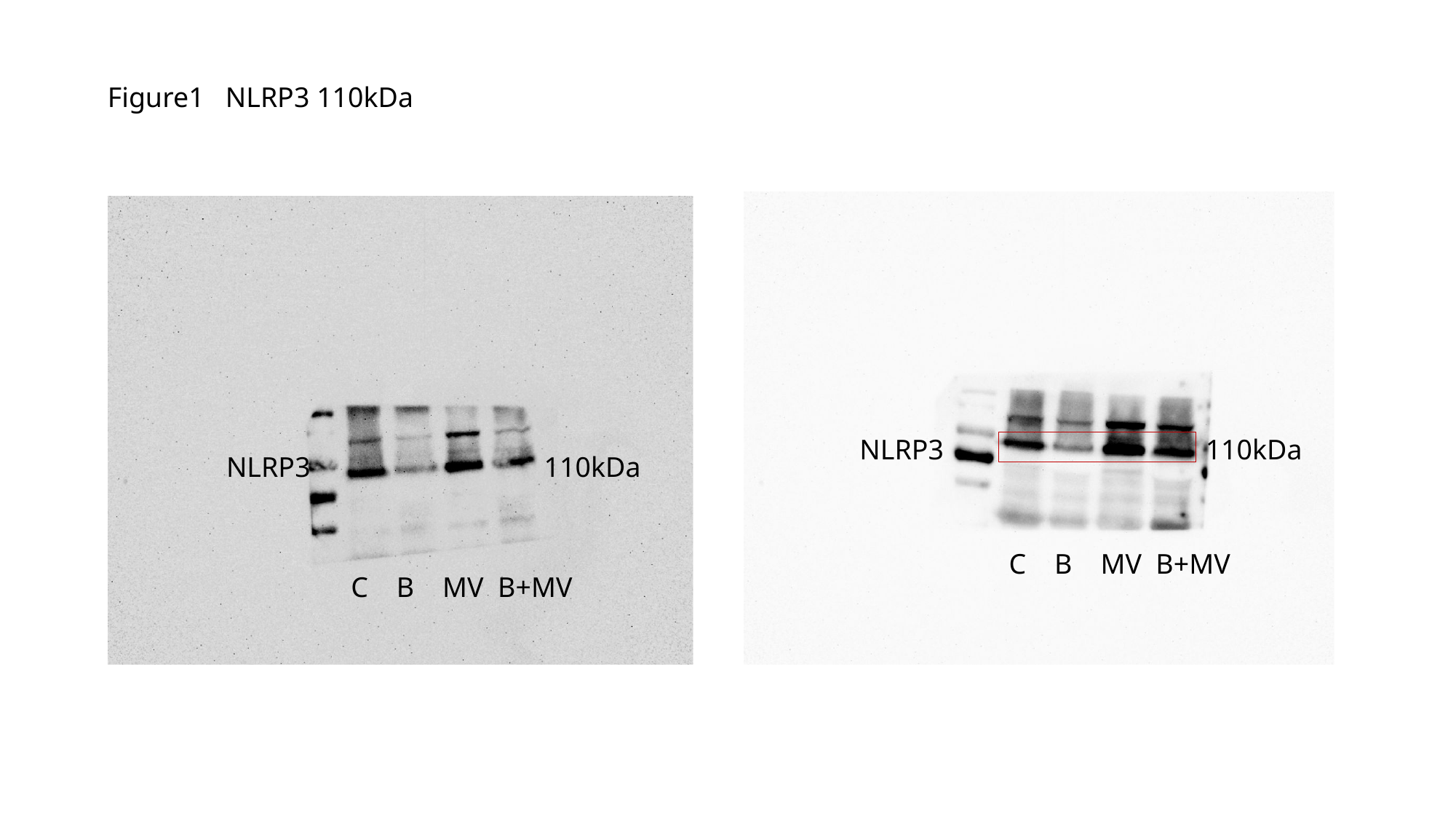

Figure1 NLRP3 110kDa
NLRP3 110kDa
NLRP3 110kDa
C B MV B+MV
 C B MV B+MV

## Slide 2
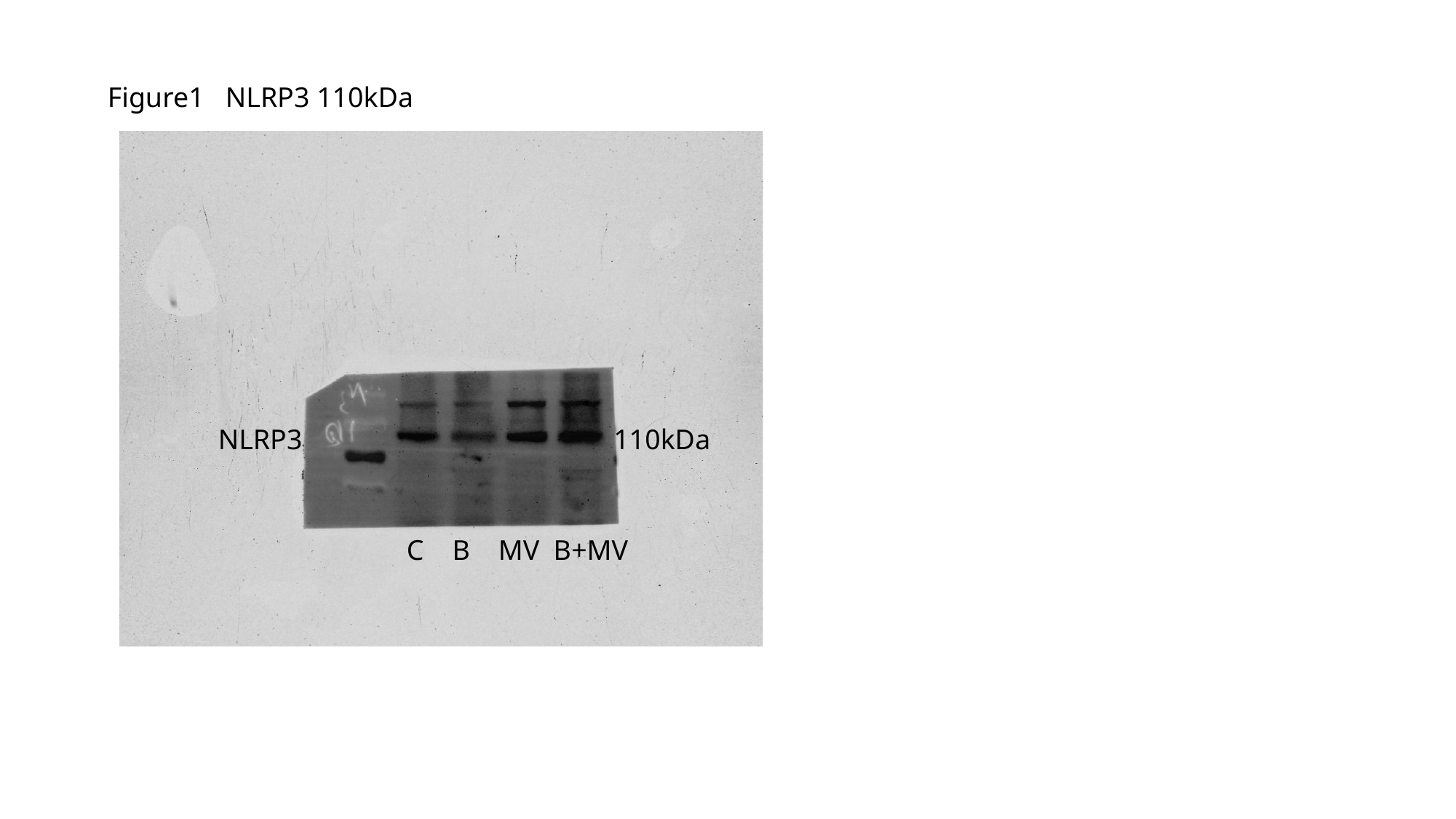

Figure1 NLRP3 110kDa
NLRP3 110kDa
 C B MV B+MV

## Slide 3
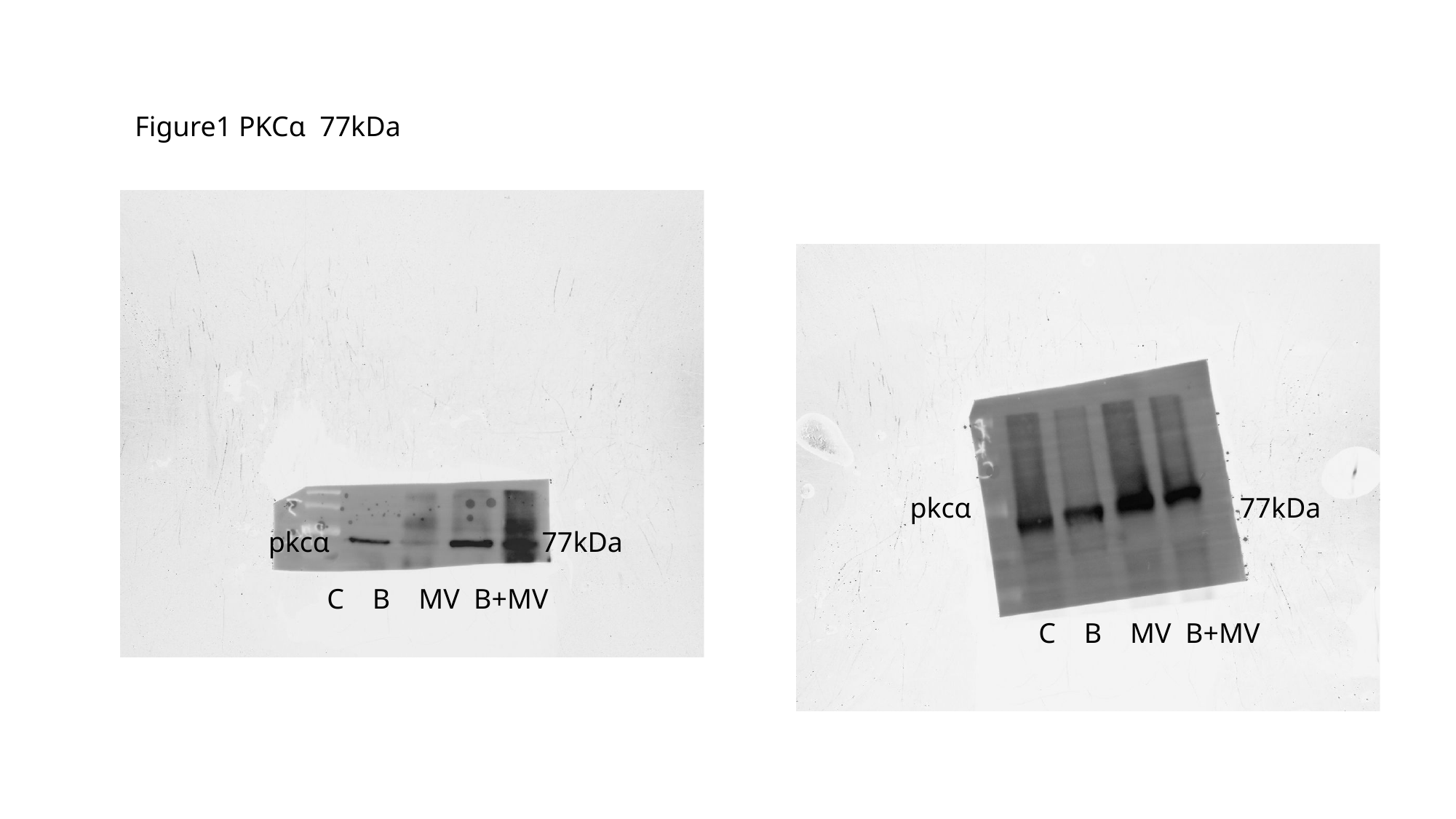

Figure1 PKCɑ 77kDa
pkcα 77kDa
pkcα 77kDa
C B MV B+MV
C B MV B+MV

## Slide 4
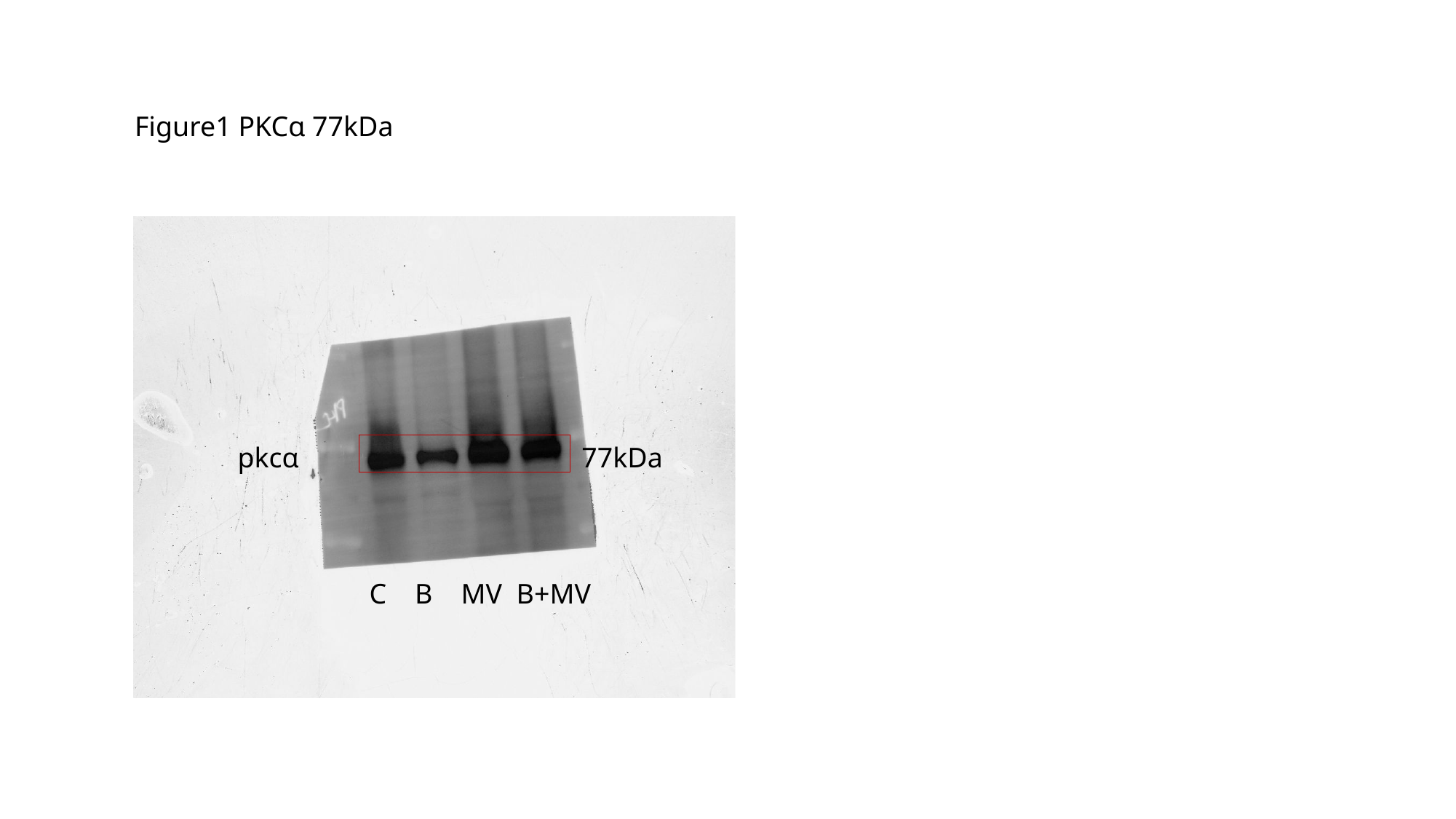

Figure1 PKCɑ 77kDa
pkcα 77kDa
C B MV B+MV

## Slide 5
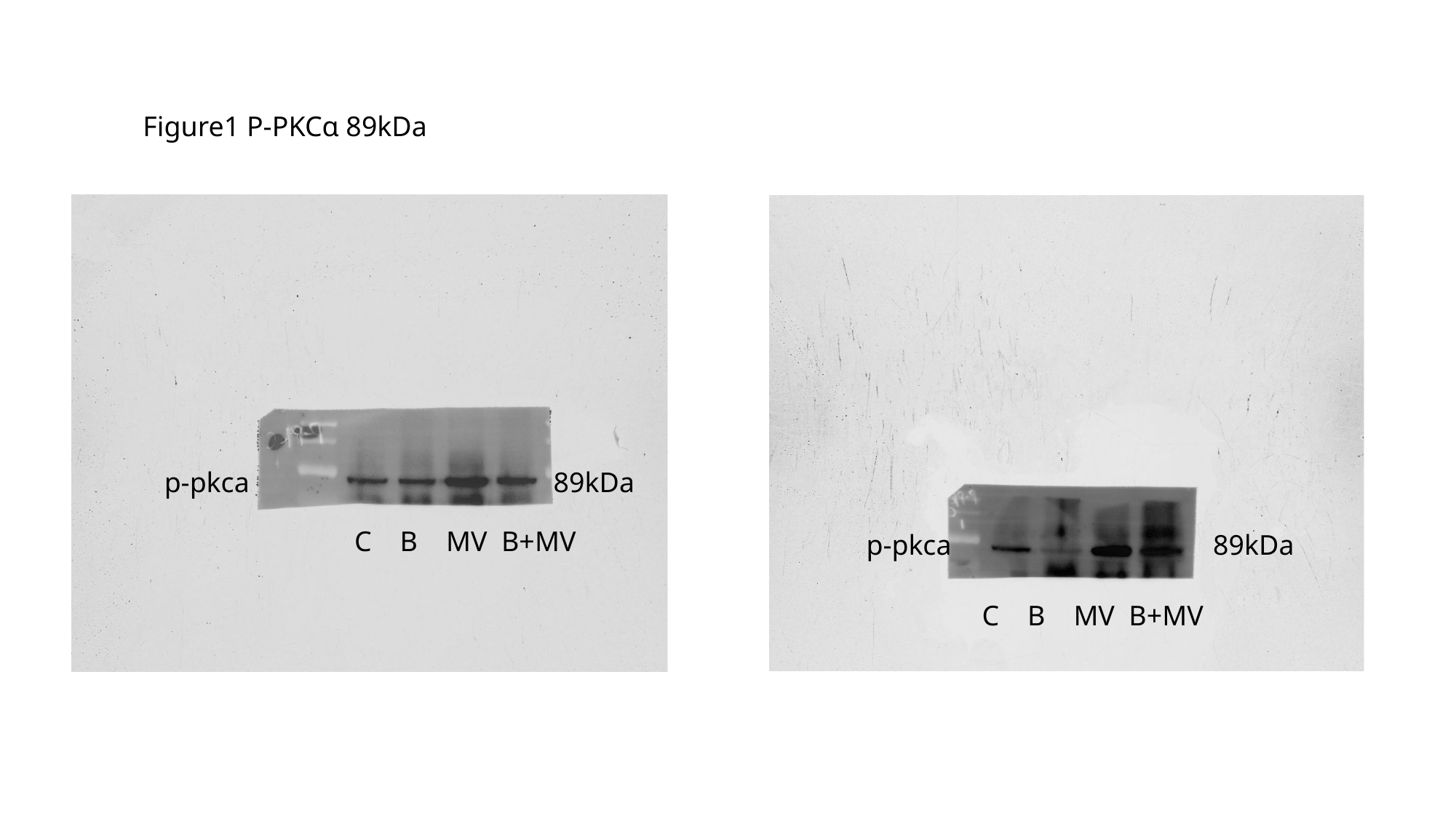

Figure1 P-PKCɑ 89kDa
p-pkca 89kDa
 C B MV B+MV
p-pkca 89kDa
 C B MV B+MV

## Slide 6
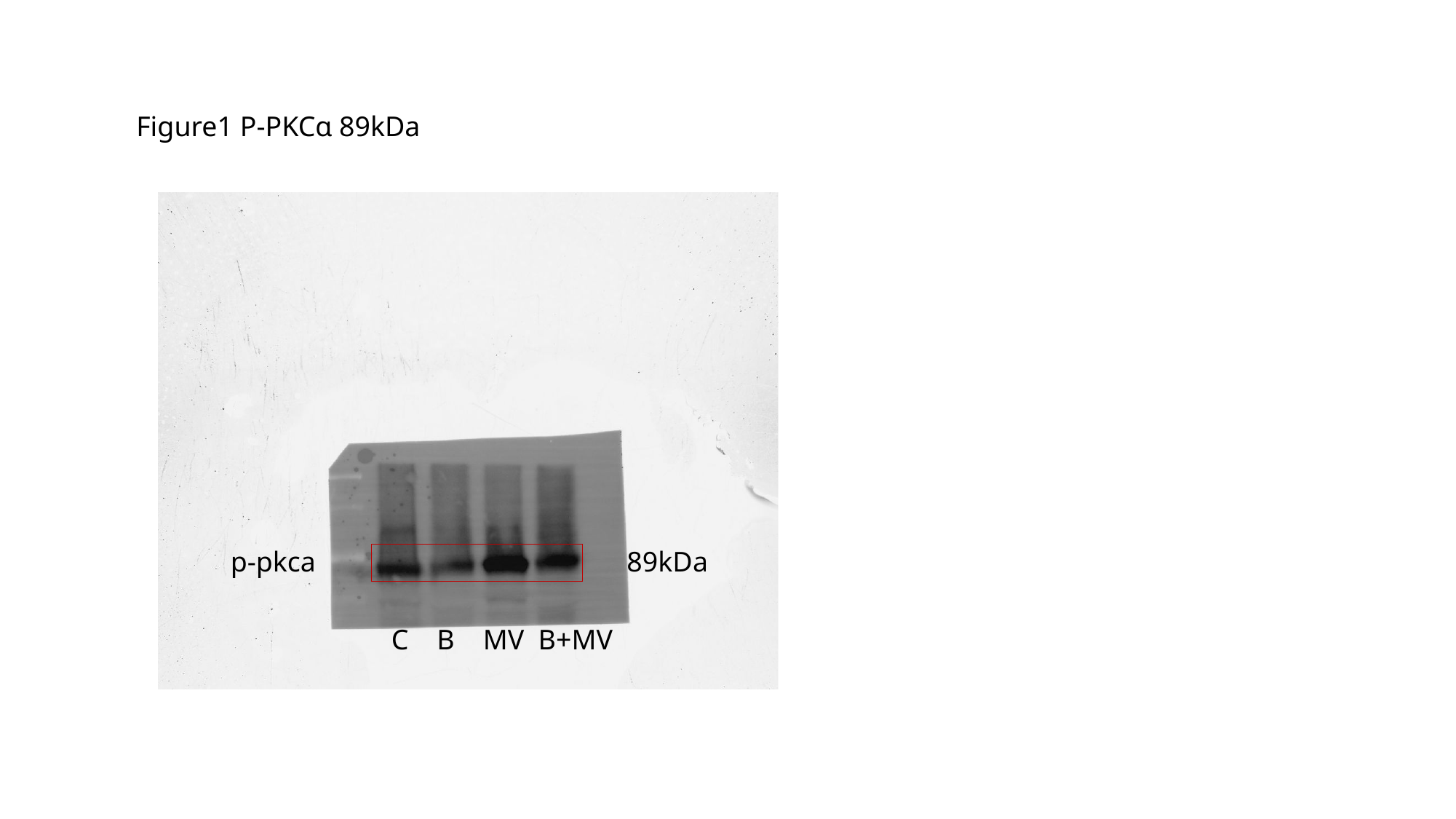

Figure1 P-PKCɑ 89kDa
p-pkca 89kDa
C B MV B+MV

## Slide 7
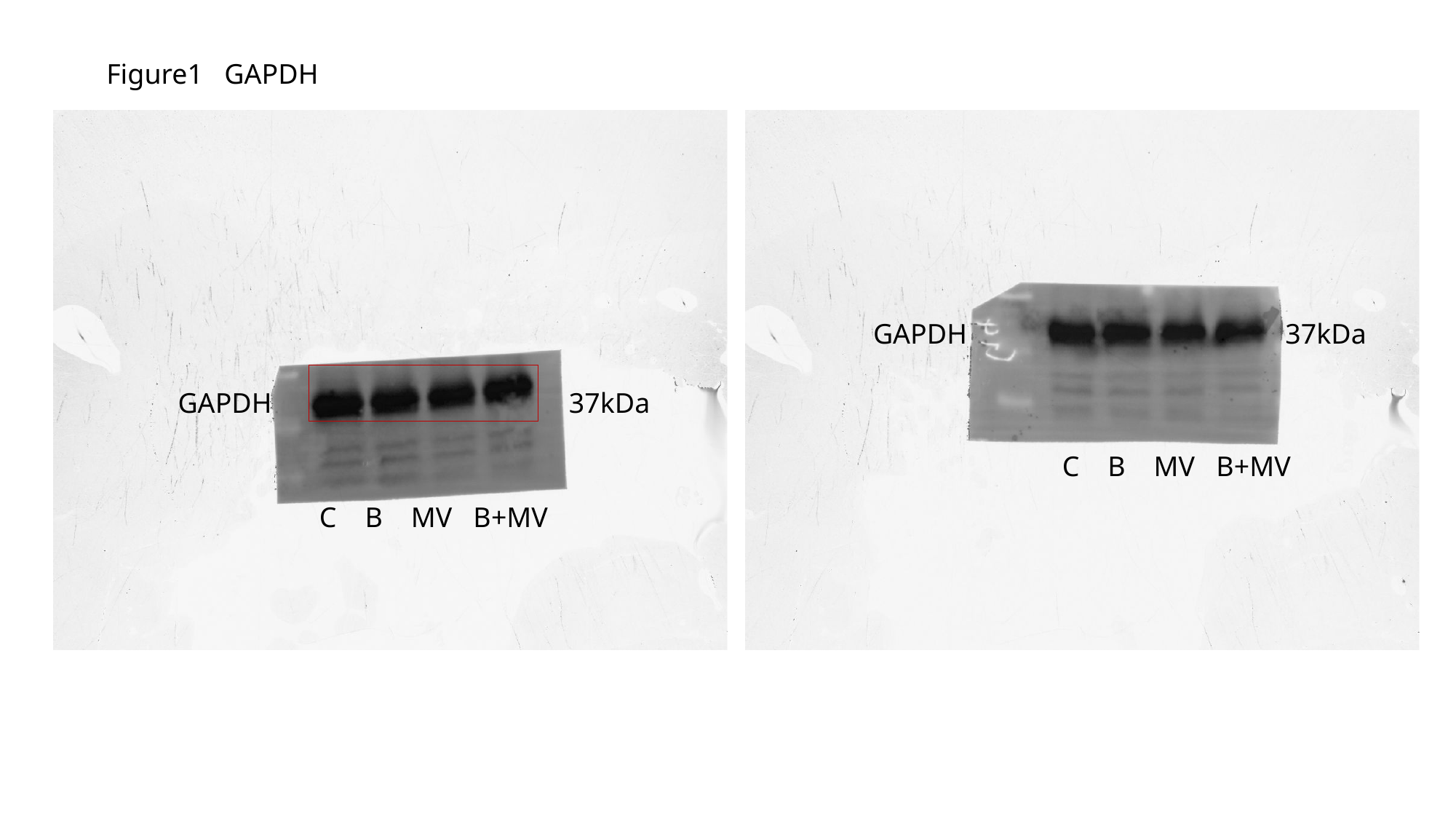

Figure1 GAPDH
GAPDH 37kDa
GAPDH 37kDa
 C B MV B+MV
 C B MV B+MV

## Slide 8
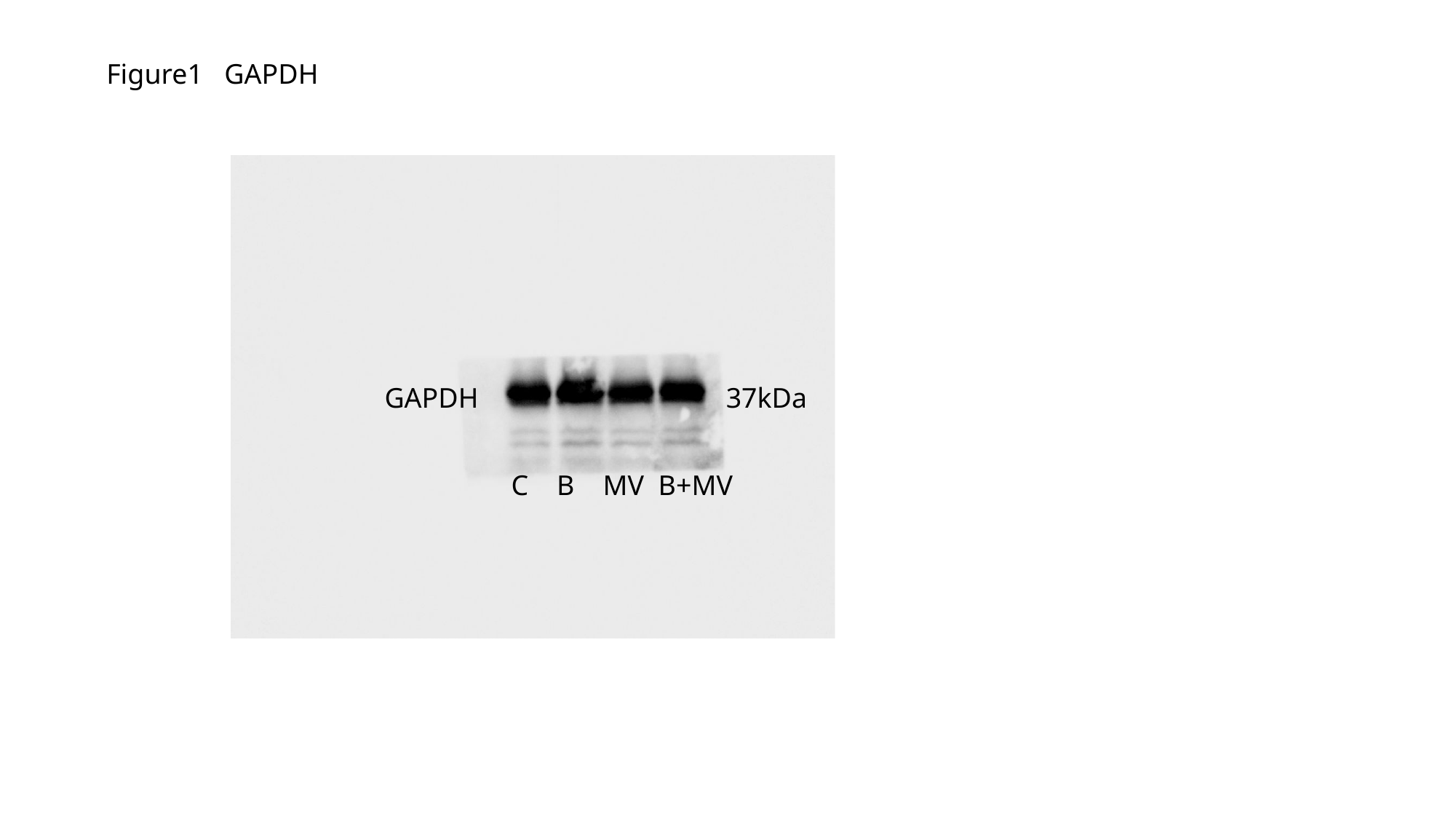

Figure1 GAPDH
GAPDH 37kDa
 C B MV B+MV

## Slide 9
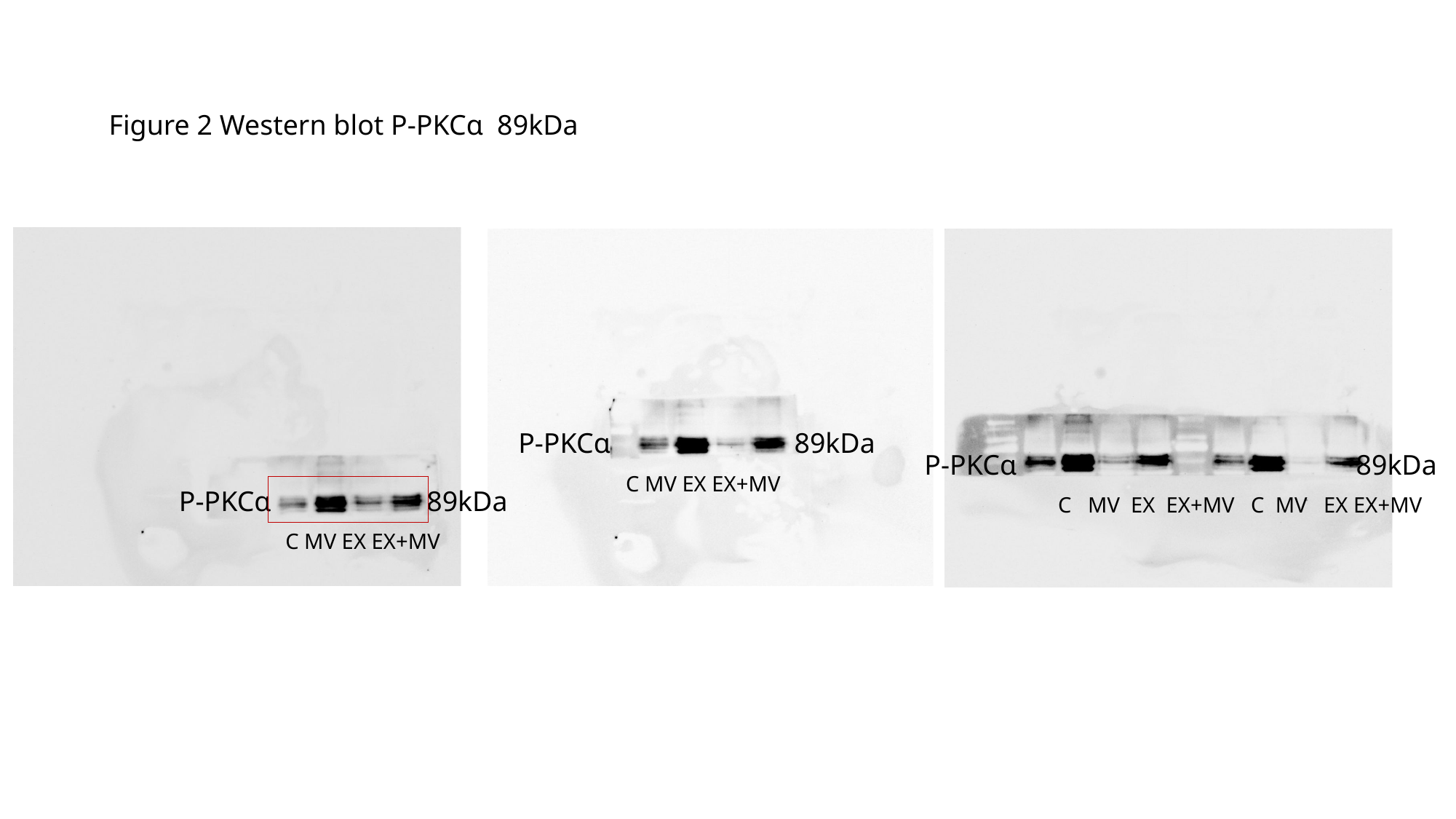

Figure 2 Western blot P-PKCɑ 89kDa
P-PKCɑ 89kDa
P-PKCɑ 89kDa
C MV EX EX+MV
P-PKCɑ 89kDa
 C MV EX EX+MV C MV EX EX+MV
C MV EX EX+MV

## Slide 10
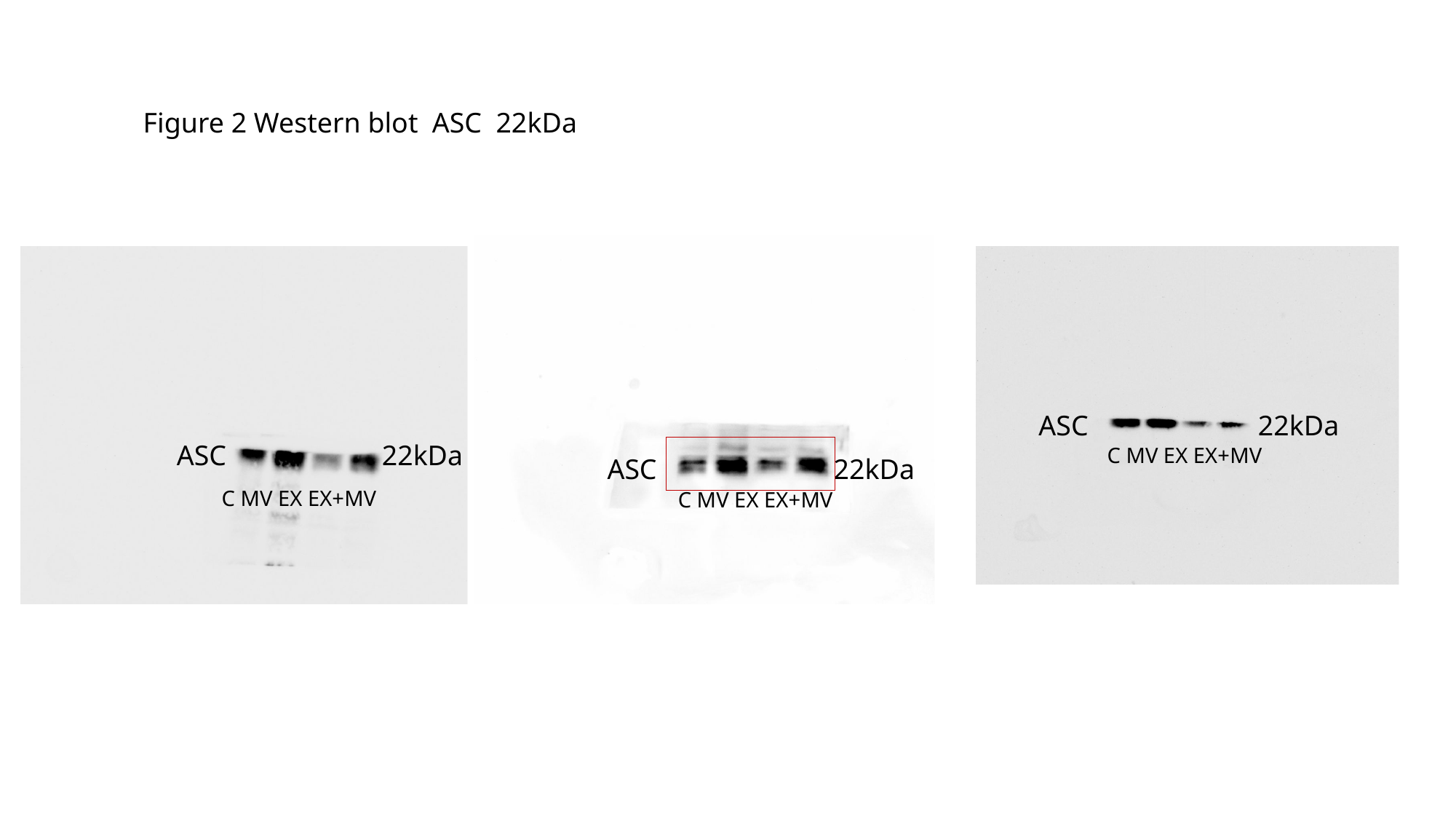

Figure 2 Western blot ASC 22kDa
ASC 22kDa
ASC 22kDa
C MV EX EX+MV
ASC 22kDa
C MV EX EX+MV
C MV EX EX+MV

## Slide 11
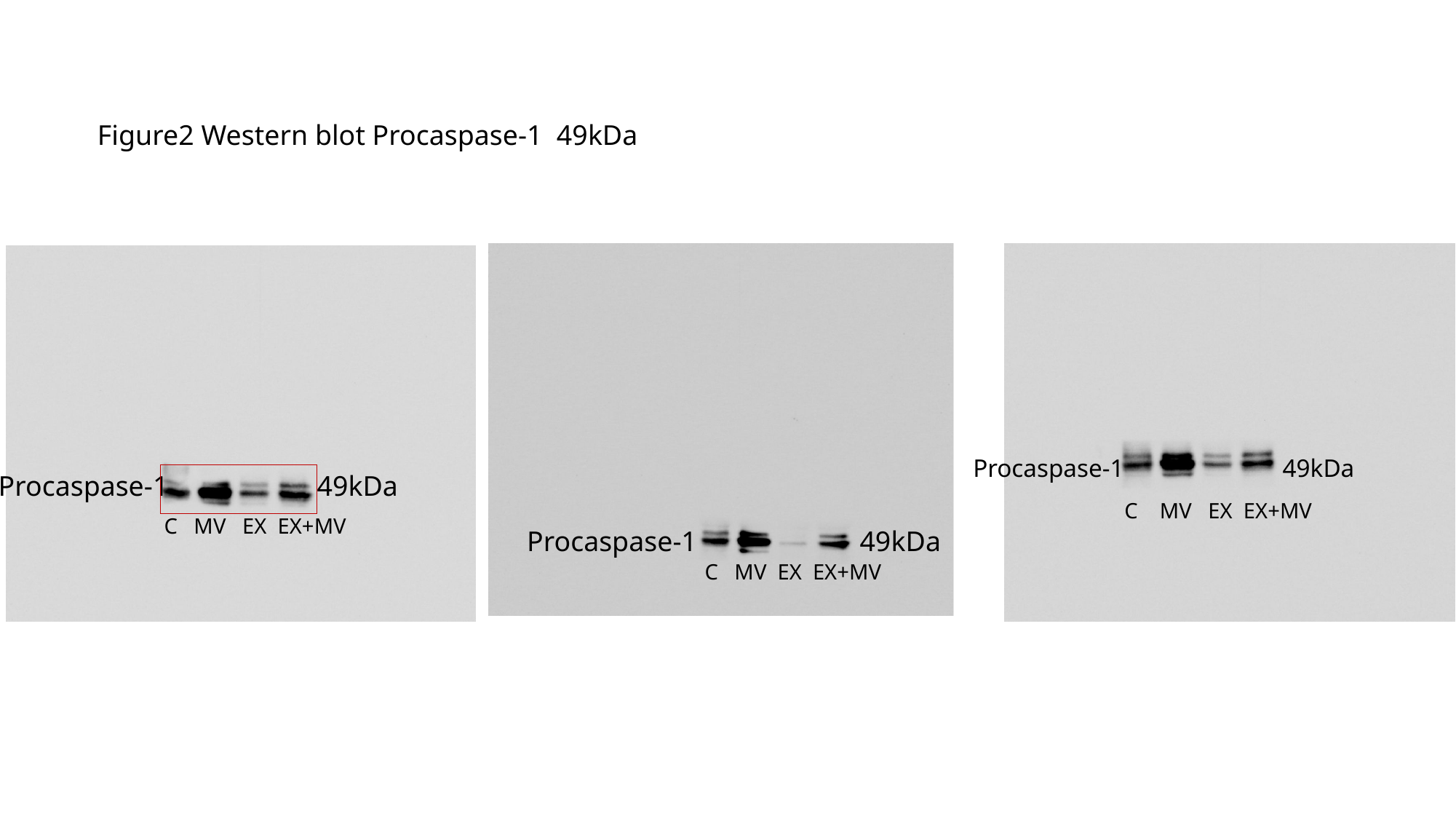

Figure2 Western blot Procaspase-1 49kDa
Procaspase-1 49kDa
Procaspase-1 49kDa
C MV EX EX+MV
C MV EX EX+MV
Procaspase-1 49kDa
 C MV EX EX+MV

## Slide 12
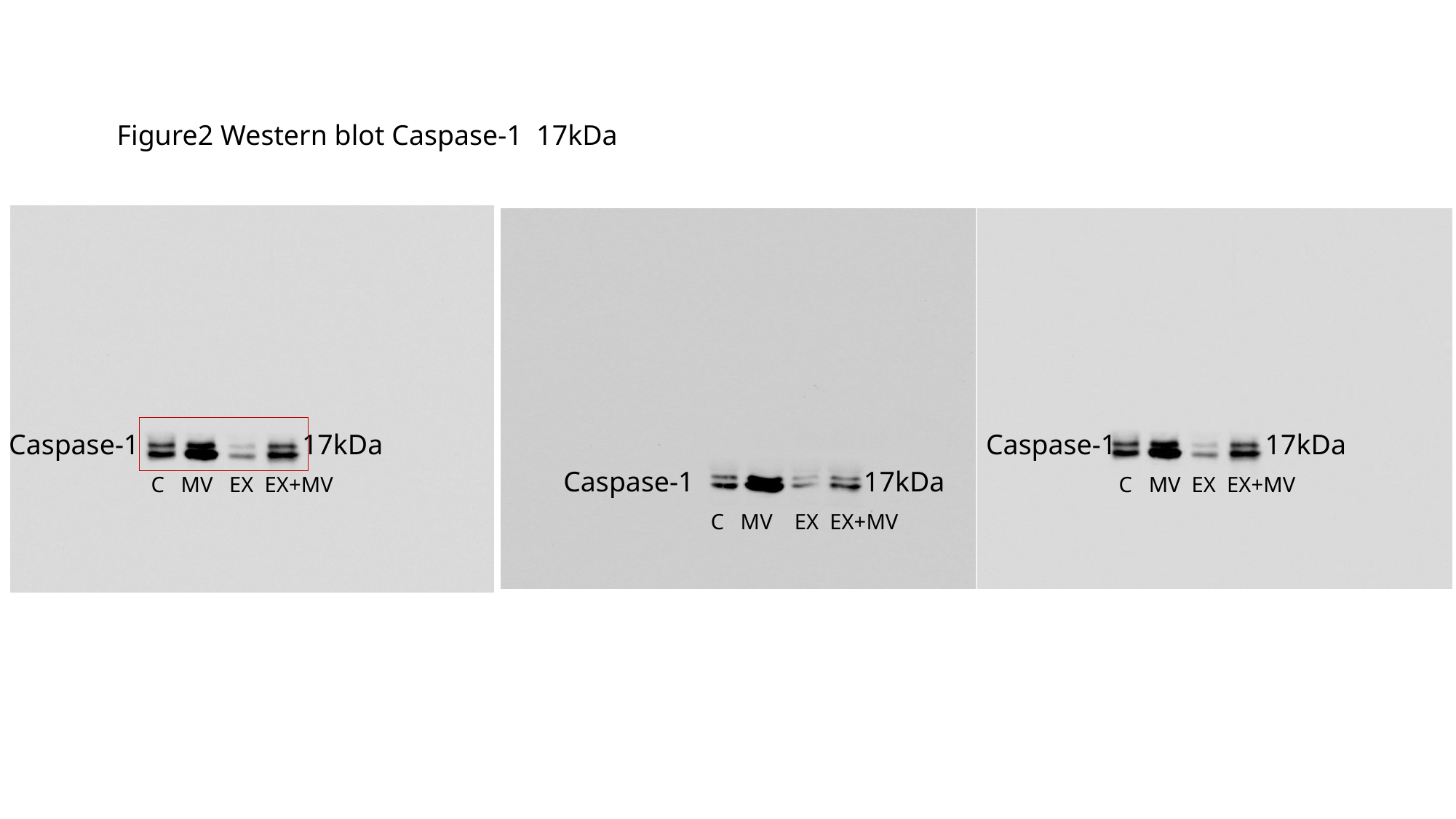

Figure2 Western blot Caspase-1 17kDa
Caspase-1 17kDa
Caspase-1 17kDa
Caspase-1 17kDa
C MV EX EX+MV
 C MV EX EX+MV
C MV EX EX+MV

## Slide 13
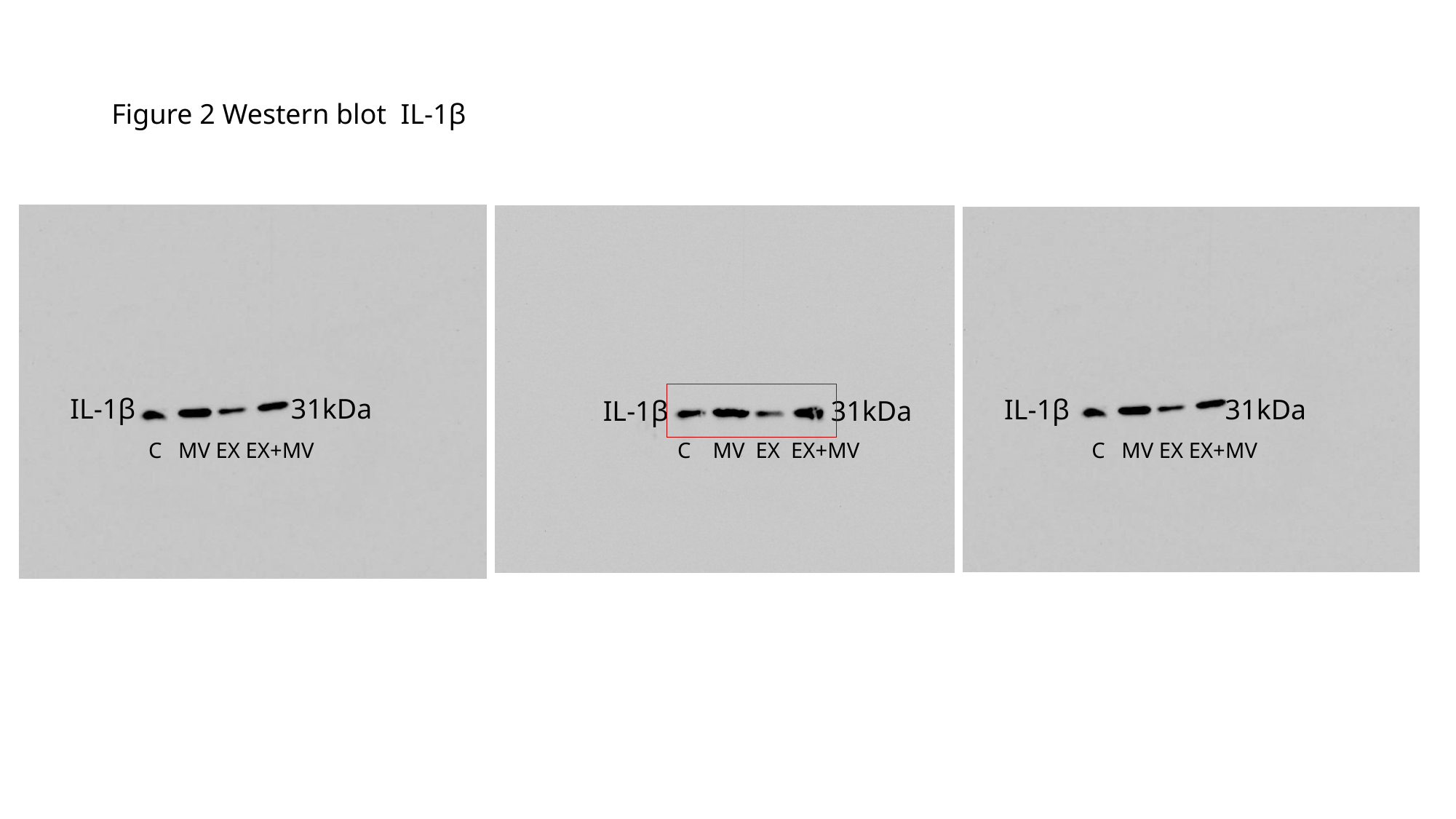

Figure 2 Western blot IL-1β
IL-1β 31kDa
IL-1β 31kDa
IL-1β 31kDa
C MV EX EX+MV
C MV EX EX+MV
C MV EX EX+MV

## Slide 14
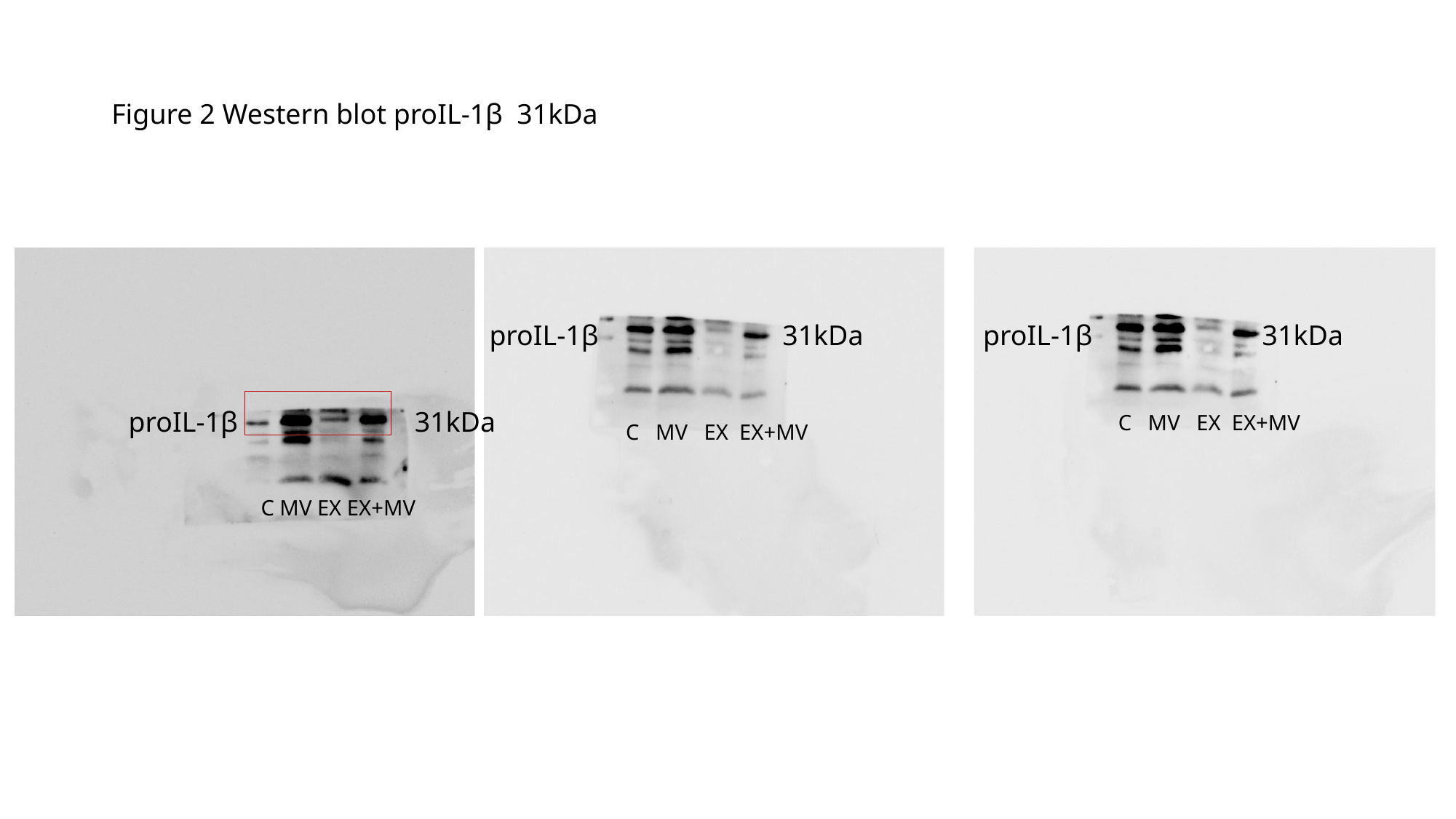

Figure 2 Western blot proIL-1β 31kDa
proIL-1β 31kDa
proIL-1β 31kDa
proIL-1β 31kDa
 C MV EX EX+MV
C MV EX EX+MV
C MV EX EX+MV

## Slide 15
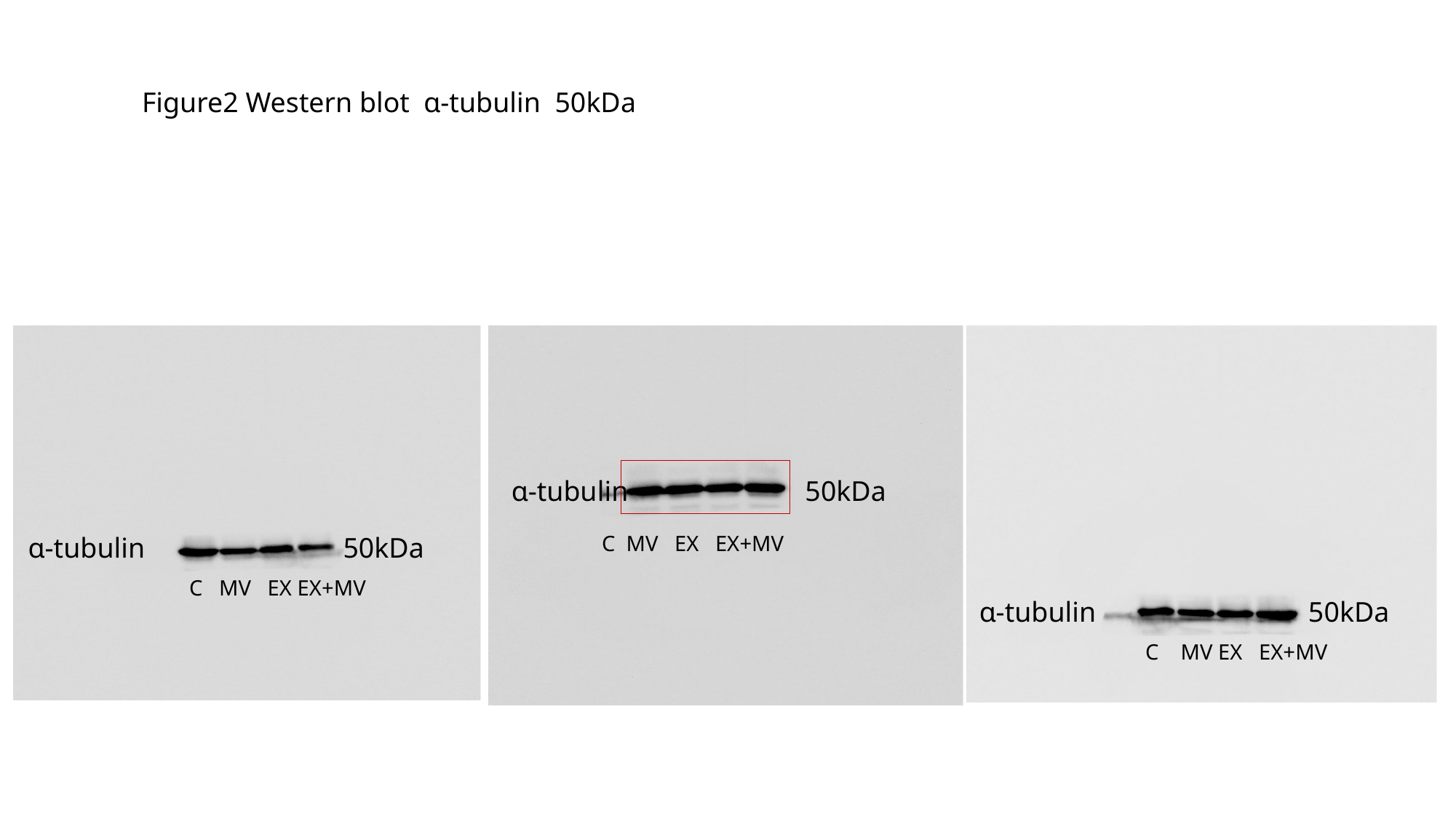

Figure2 Western blot ɑ-tubulin 50kDa
ɑ-tubulin 50kDa
ɑ-tubulin 50kDa
 C MV EX EX+MV
 C MV EX EX+MV
ɑ-tubulin 50kDa
 C MV EX EX+MV

## Slide 16
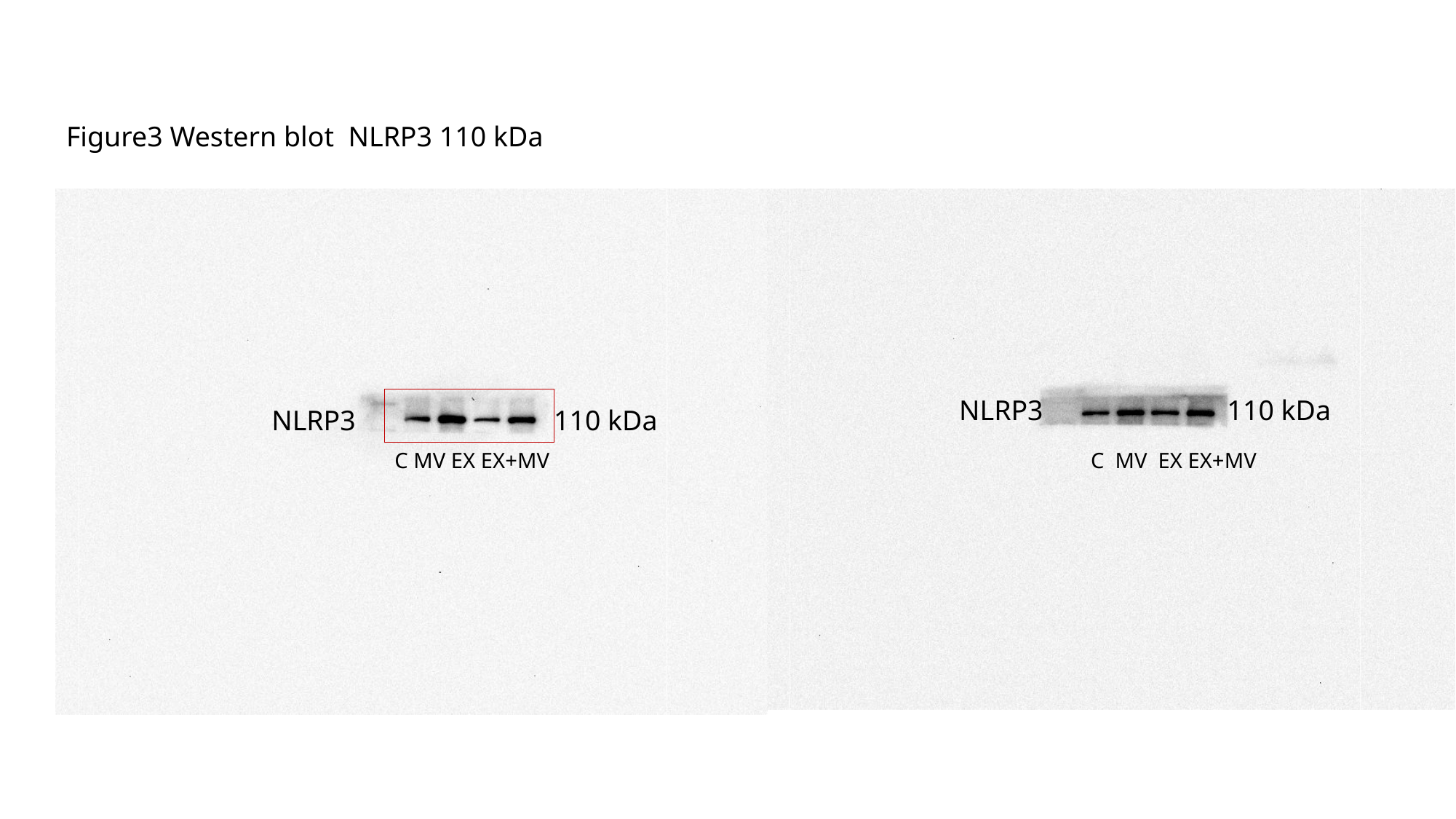

Figure3 Western blot NLRP3 110 kDa
NLRP3 110 kDa
NLRP3 110 kDa
C MV EX EX+MV
 C MV EX EX+MV

## Slide 17
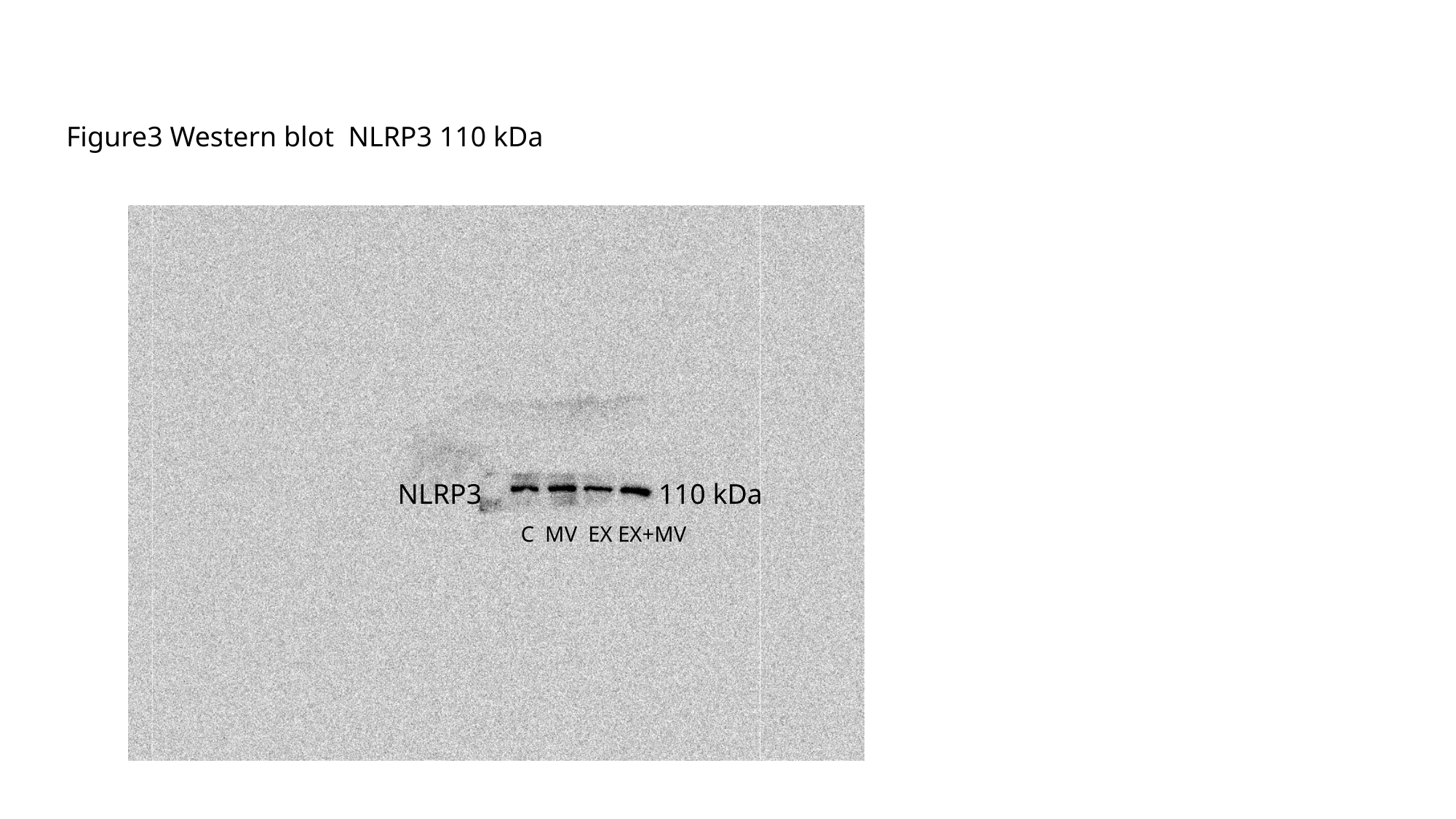

Figure3 Western blot NLRP3 110 kDa
NLRP3 110 kDa
 C MV EX EX+MV

## Slide 18
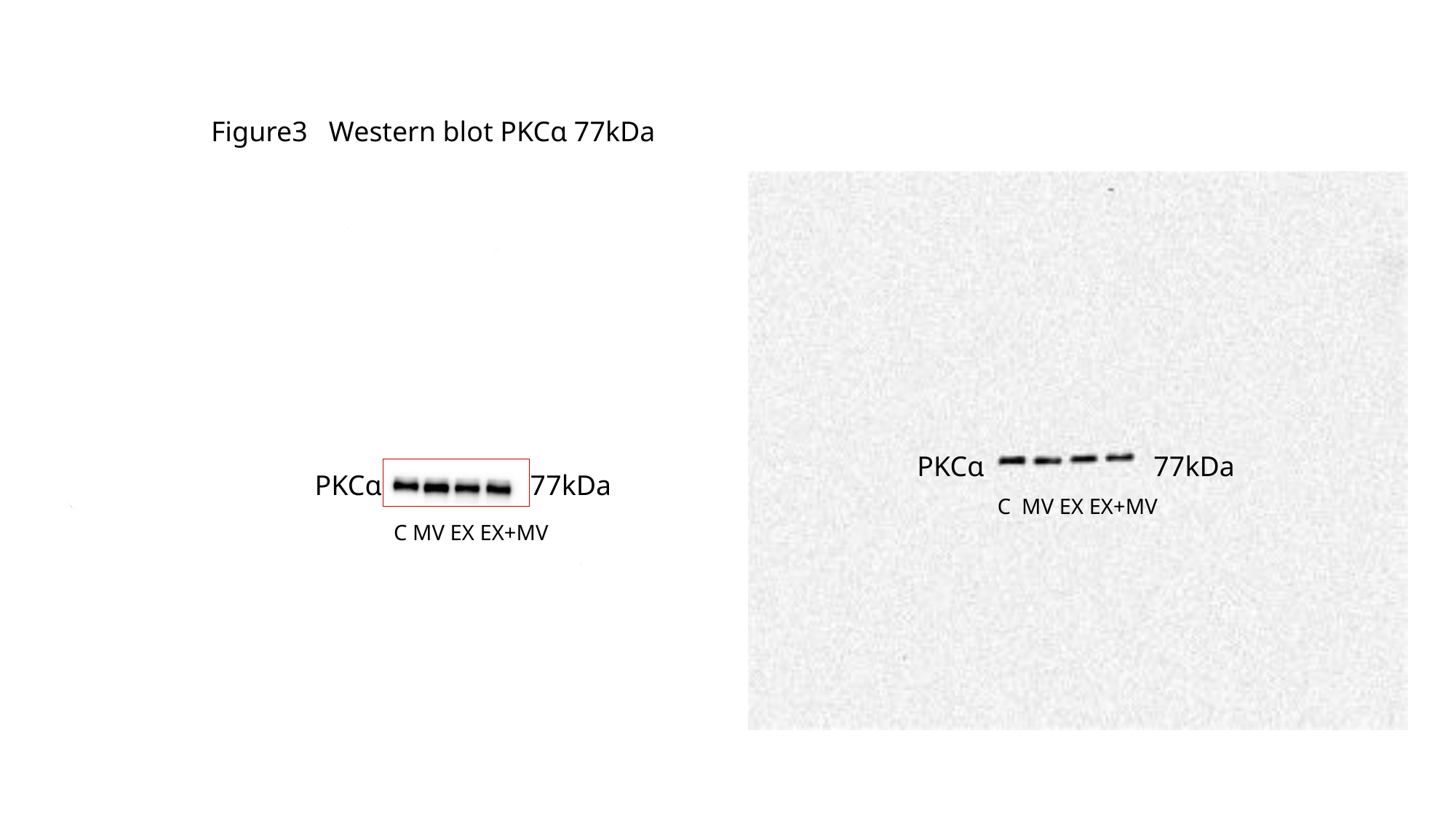

Figure3 Western blot PKCɑ 77kDa
PKCɑ 77kDa
PKCɑ 77kDa
C MV EX EX+MV
C MV EX EX+MV

## Slide 19
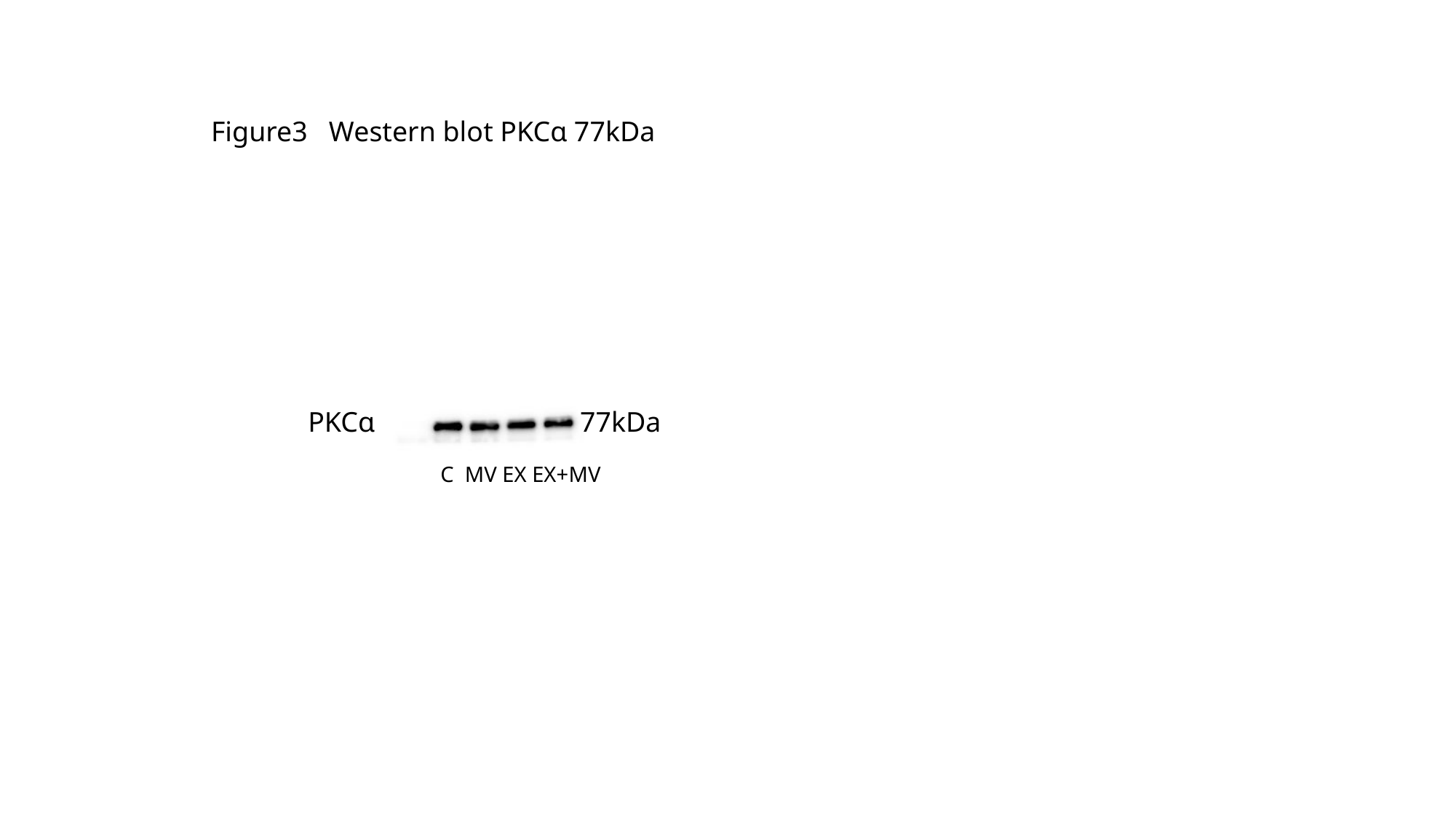

Figure3 Western blot PKCɑ 77kDa
PKCɑ 77kDa
C MV EX EX+MV

## Slide 20
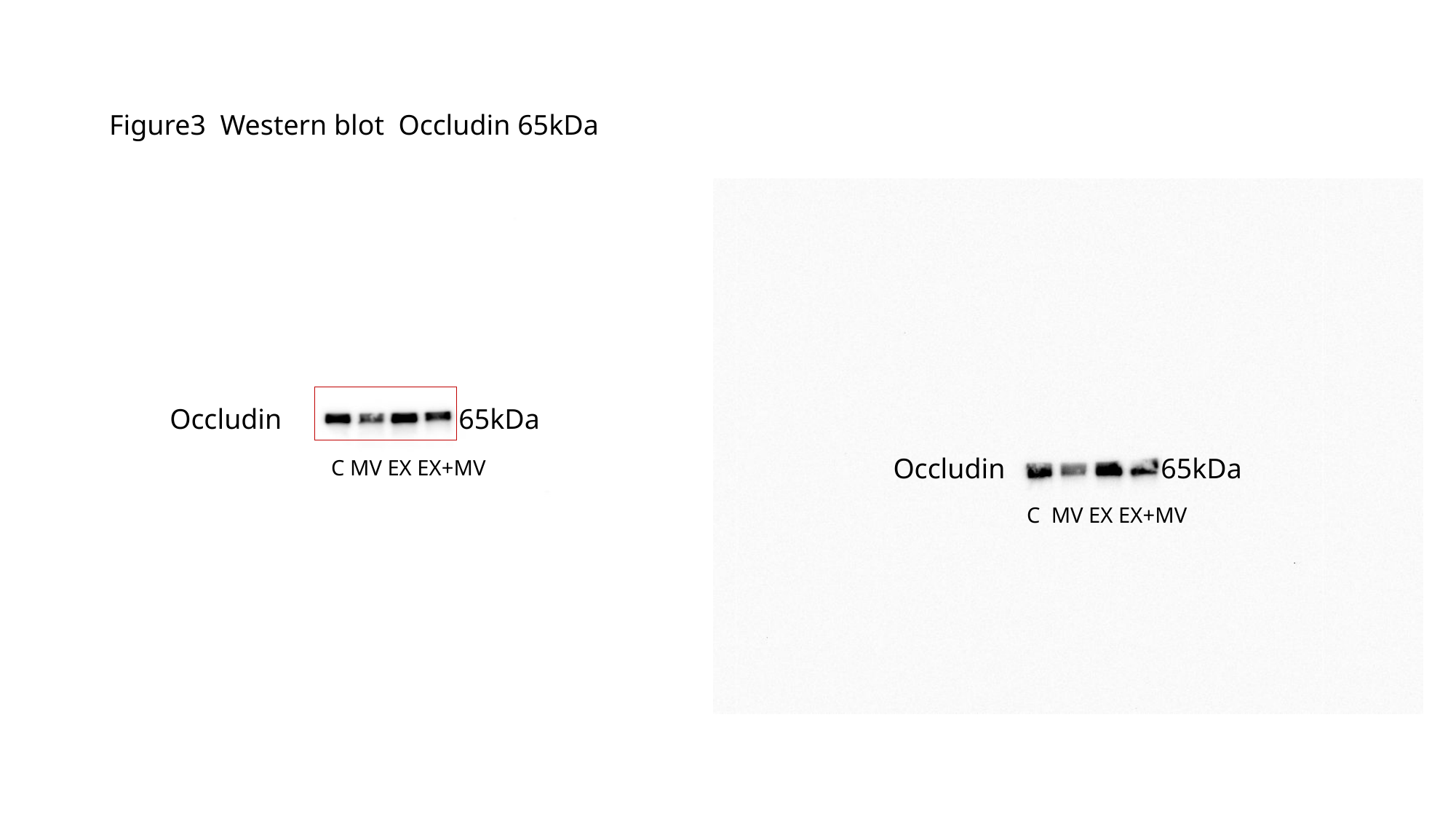

Figure3 Western blot Occludin 65kDa
Occludin 65kDa
Occludin 65kDa
 C MV EX EX+MV
C MV EX EX+MV

## Slide 21
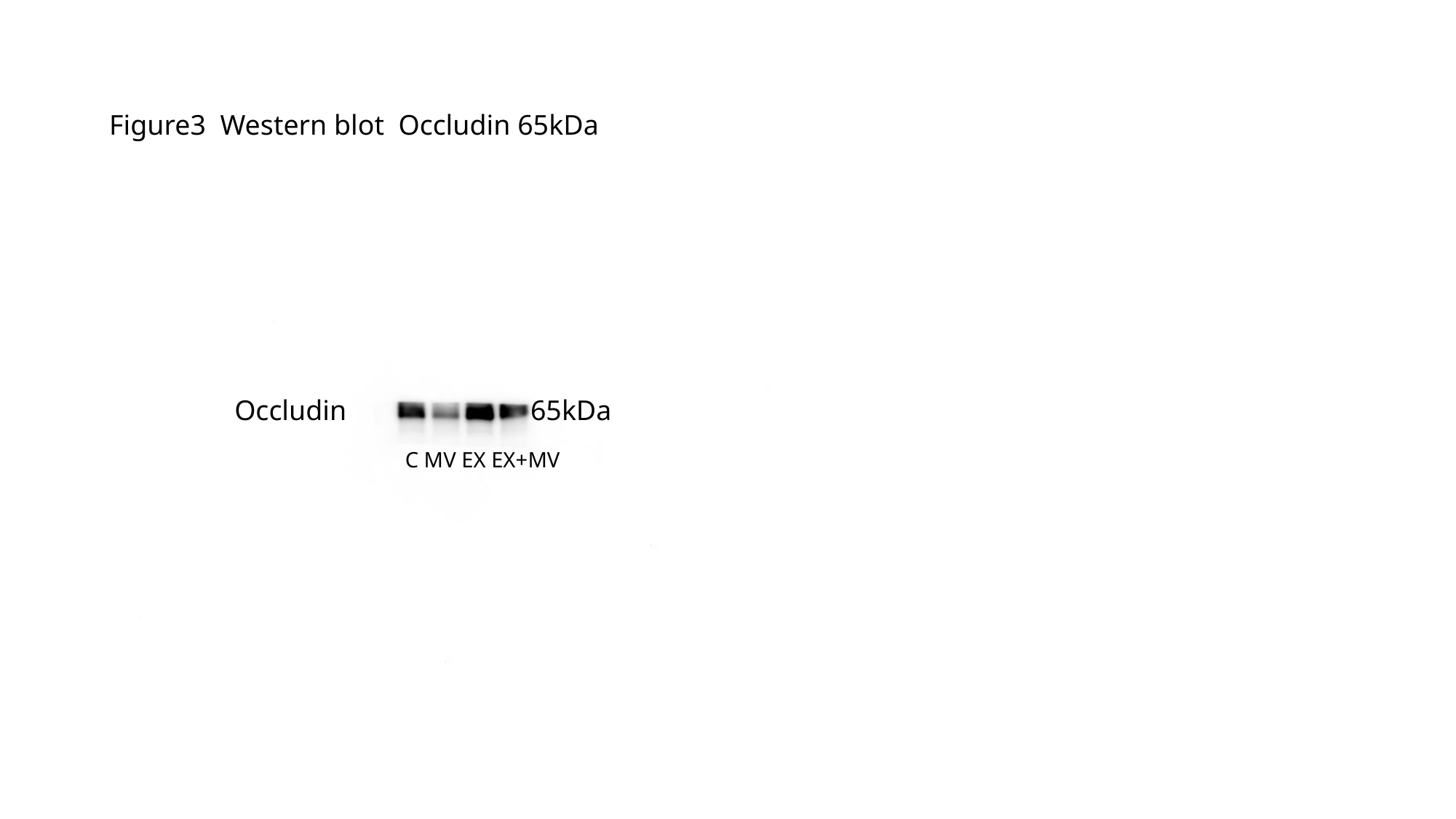

Figure3 Western blot Occludin 65kDa
Occludin 65kDa
 C MV EX EX+MV

## Slide 22
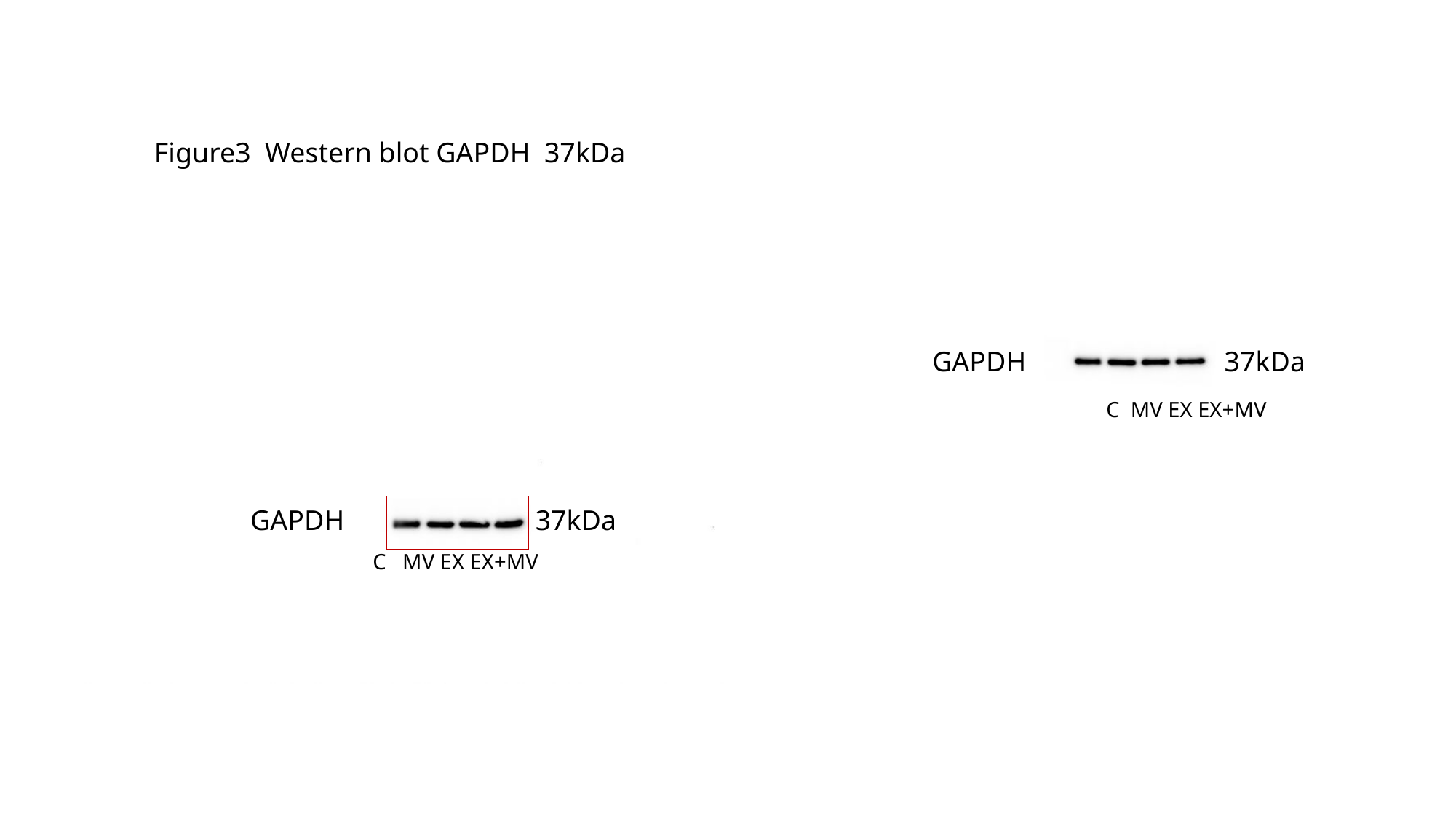

Figure3 Western blot GAPDH 37kDa
GAPDH 37kDa
C MV EX EX+MV
GAPDH 37kDa
C MV EX EX+MV

## Slide 23
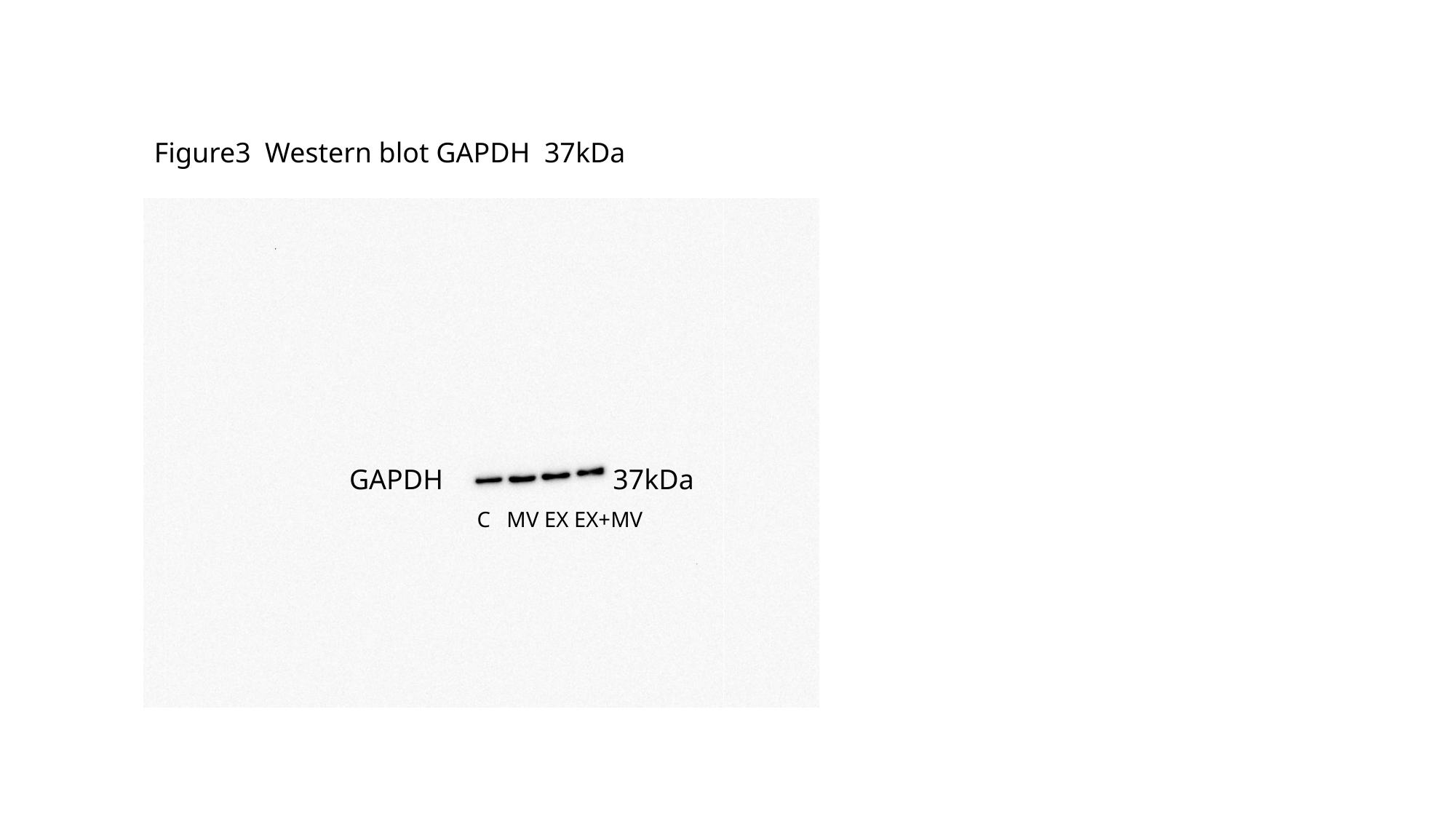

Figure3 Western blot GAPDH 37kDa
GAPDH 37kDa
 C MV EX EX+MV
